# A high-throughput optomechanical retrieval method for sequence-verified clonal DNA from the NGS platform

**DOI:** 10.1038/ncomms7073

**Published:** 2015-02-02

**Authors:** Howon Lee, Hyoki Kim, Sungsik Kim, Taehoon Ryu, Hwangbeom Kim, Duhee Bang, Sunghoon Kwon

**Affiliations:** 1 Institutes of Entrepreneurial BioConvergence, Seoul National University, Seoul 151-742, Republic of Korea; 2 Celemics Inc., 371-17, Gasan-dong, Geumcheon-gu, Seoul 153-718, Republic of Korea; 3 Interdisciplinary Program for Bioengineering, Seoul National University, Seoul 151-744, Republic of Korea; 4 Department of Electrical Engineering and Computer Science, Seoul National University, Seoul 151-744, Republic of Korea; 5 Department of Chemistry and Biochemistry, University of California, Los Angeles, California 90095-1569, USA; 6 Department of Chemistry, Yonsei University, Seoul 120-749, Republic of Korea; 7 Center for Nanoparticle Research, Institute for Basic Science, Seoul National University, Seoul 151-742, Republic of Korea; 8 Seoul National University Hospital Biomedical Research Institute 101 Daehakro, Chungrogu, Seoul National University Hospital, Seoul 110-744, Republic of Korea

## Abstract

Writing DNA plays a significant role in the fields of synthetic biology, functional genomics and bioengineering. DNA clones on next-generation sequencing (NGS) platforms have the potential to be a rich and cost-effective source of sequence-verified DNAs as a precursor for DNA writing. However, it is still very challenging to retrieve target clonal DNA from high-density NGS platforms. Here we propose an enabling technology called ‘Sniper Cloning’ that enables the precise mapping of target clone features on NGS platforms and non-contact rapid retrieval of targets for the full utilization of DNA clones. By merging the three cutting-edge technologies of NGS, DNA microarray and our pulse laser retrieval system, Sniper Cloning is a week-long process that produces 5,188 error-free synthetic DNAs in a single run of NGS with a single microarray DNA pool. We believe that this technology has potential as a universal tool for DNA writing in biological sciences.

## Abstract (web summary)

One of the biggest bottlenecks in large-scale DNA synthesis is the retrieval of target clonal DNA from high-density sequencing platforms. Here, the authors present a method called ‘Sniper Cloning’ that allows for precise mapping of target clone features and rapid retrieval of targets for full utilization of DNA clones.

## Graphical abstract

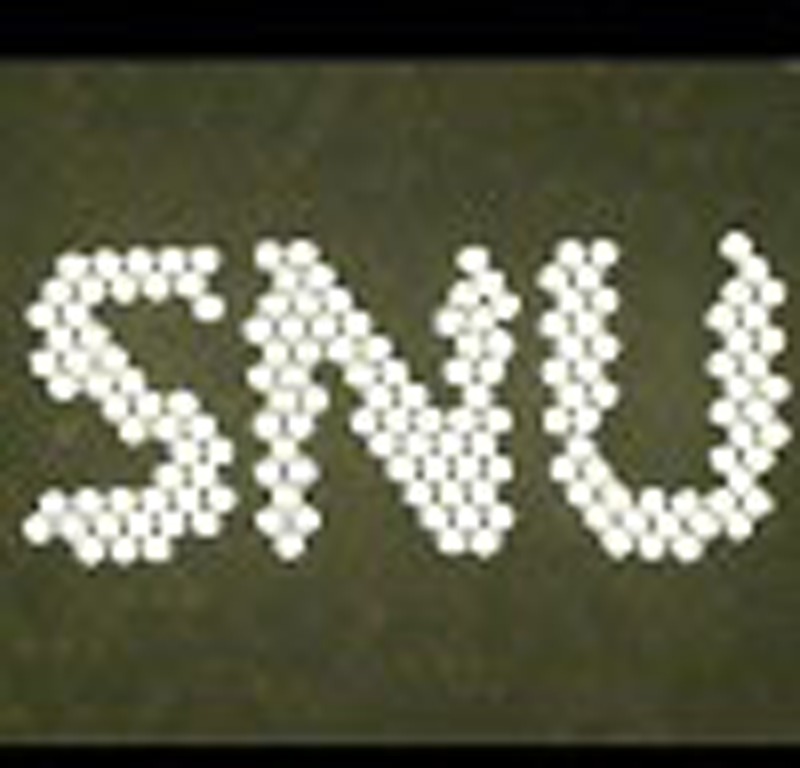

Writing long DNA requires piece-wise bottom-up assembly of numerous high-purity oligonucleotides as building blocks because of the current limitation of coupling efficiency (<99.5%) in solid-phase synthesis of oligonucleotides^1^,^2^,^3^,^4^. However, the cost of writing long DNA such as bacterial genome DNA (>1 Mb) is over one million dollars, of which the greatest expense is the synthesis and purification of precursor building blocks (Supplementary Fig. 1). Recent innovations in parallel synthesis technologies using DNA microarrays offer a mixed pool of millions of distinct nucleotides (~200 nt) in a single run at a cost a hundred times lower than that of individual solid-phase synthesis^5^,^6^,^7^,^8^,^9^. In spite of the significant throughput enhancement in the synthesis process, selecting the correct nucleotides from the highly mixed and complex pool of error-prone oligonucleotides severely increases the expense and obscures the major benefits of microarray synthesis.

Over the past 10 years, various approaches have been proposed to reduce the complexity of microarray-derived mixed pools of DNA, but the appropriate selection of error-free DNA segments is still difficult. Conventional *in vivo* cloning has little utility in the purification of the complex pool of oligonucleotides because of the insufficient throughput of fully randomized pick-and-place colony selection followed by Sanger sequencing (Supplementary Note 1)^10^. Recent studies incorporated the high-throughput analysis capacity of next-generation sequencing (NGS)^11^,^12^,^13^,^14^,^15^,^16^,^17^. The use of specific barcoded primer pairs enables the retrieval of NGS-verified target species from the microarray-derived oligonucleotide mixture by selective amplification^18^,^19^. However, retrieval through random barcode amplification potentially results in a considerable proportion of missing or mixed sequences due to the complexity of pool and primer contents and the formation of hairpins or dimers^20^. The extra processing required to reduce the pool complexity, the specific design rules for primer pairs to maintain orthogonality, the non-uniform PCR products and the great expense of preparing the huge numbers of barcode primer pairs needed to deal with a high proportion of error-carrying species in microarray DNA cannot be ignored in larger-scale experiments. In 2010, Matzas *et al*. introduced NGS technology as a novel high-throughput purification method for microarray-derived oligonucleotides^21^. Their approach involved the selection of sequenced DNA clones one at a time by physically contacting DNA beads. This approach cleverly turns a used sequencing plate, which is typically discarded after sequencing, into an ultra-rich source of sequence-verified DNA. Although NGS could potentially be used for the purification of a highly complex mixture of DNAs from a microarray, the extremely low throughput of picking DNAs from the plates hinders the practical use of this technology. It is very challenging to retrieve purified clonal DNA from the high-density NGS platforms because of the very small size (~30 μm) of clonal beads and poorly-defined positional information. Thus, the throughput and accuracy of the conventional pick-and-place retrieval approach based on physical contact is not able to meet the demand (~10^4^ building blocks) of current megabase-sized DNA research.

Here, we introduce an enabling technology called ‘Sniper Cloning’ that enables the precise mapping of target clone features and the rapid retrieval of the target by shooting a small laser pulse onto the targeted spot. This approach has been applied to the current 454 NGS system that involves microstructure (microbeads) and is potentially applicable for higher-capacity NGS platforms with direct attachment of DNA clusters on the surface of a sequencing substrate such as Illumina (Supplementary Figs 2–9). In this way we can fully utilize the DNA clones from the NGS platforms, resulting in a cost-effective high-throughput selection of the correct building blocks for ‘writing’ DNA based on two technical breakthroughs. We develop a ‘diffusion-like local mapping algorithm’ to precisely map the target clone locations. A custom-made pulse laser optomechanical device uses radiation pressure to transfer the target clones from the microscale NGS substrate to users in a high-throughput and non-contact manner. By merging the three cutting-edge technologies of NGS, DNA microarray and our laser retrieval system, Sniper Cloning is a week-long process that produces 5,188 error-reduced synthetic DNAs in a single run of NGS with a single microarray DNA pool, which is equivalent to the output from 60,000 rounds of conventional *in vivo* clonal selection.

## Results

### Optomechanical retrieval system

**Figure 1:**
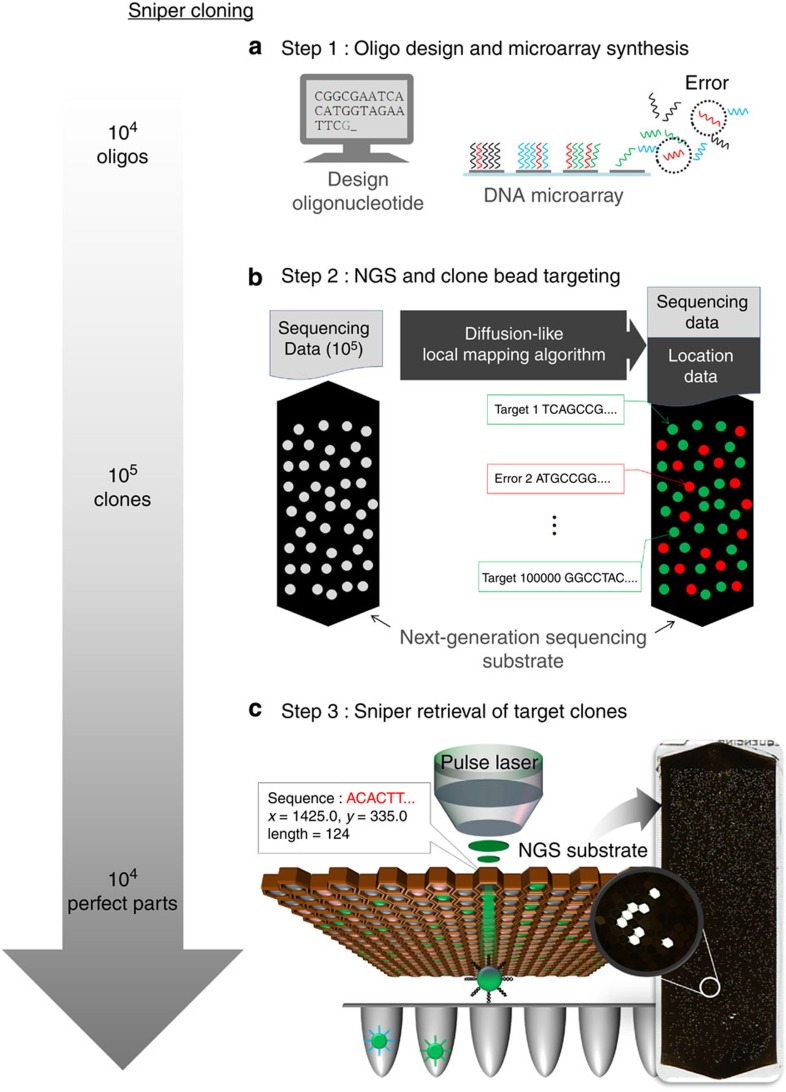
Sniper targeting of molecular clones. (**a**) Parallel synthesis of custom oligonucleotides. Constructed sequences are cleaved from the substrate forming a mixed pool of >10^4^ kinds of oligonucleotides with a certain frequency of errors (red). (**b**) NGS-based massively parallel identification was followed by clone bead location targeting using a diffusion-like local mapping algorithm. NGS enables the isolation and amplification of single molecules from a complex pool to supply a huge amount of sequencing data (10^5^) with accordant pixel information. Our diffusion-like local mapping algorithm overcomes the random and non-linear distortion of the sequencer’s imaging system and converts the pixel information into a real-world location of the target clone bead. (**c**) Sniper retrieval of target clone beads. Precise location data from the clone bead targeting step and non-contact pulse laser bead retrieval system enable high-throughput (two beads per second) selective separation of 10^4^ perfect regions without cross-contamination. The integrated process of parallel synthesis (DNA microarray), parallel identification (NGS) and high-throughput separation (pulse laser optical retrieval system) dramatically reduces the necessary resources by eliminating the randomness of the conventional cloning method; this provides a huge amount of ultra-high quality artificial oligonucleotides within several days.

‘Sniper’ retrieval of target clones on a NGS substrate is achieved by setting up a closely integrated technical procedure including DNA microarray, NGS, and a pulse laser retrieval system. DNA microarrays synthesize >10,000 short (120 nt) single-stranded oligonucleotides with a certain frequency of errors (Fig. 1a). The NGS platform GS Junior from Roche 454 Life Sciences identifies the content of the complex pool of DNAs from the microarray through *in vitro* cloning followed by massively parallel pyrosequencing. We developed a ‘diffusion-like local mapping algorithm’ to pinpoint the exact location of the target clone beads on the substrate, and selectively separated the beads containing the desired sequence-verified oligonucleotides for direct utilization (Fig. 1b). We used the radiation pressure^22^,^23^ of a focused pulse laser to retrieve the target beads from the microscale sequencing substrate for delivery to the macro-world (Fig. 1c). The non-contact nature of light potentially reduces the possibility of cross-contamination, which is frequently induced by physical contact with micro tweezers or tips. In our approach, additional washing and replacement of physical equipment are not required. Also, with the help of an automated linear motorized stage, the high precision of the focused pulse laser provides accurate targeting of the desired molecular clones with minimal variation, enabling high-throughput retrieval (two beads per second) that is orders of magnitude faster than that of the contact approach (Supplementary Movie 1). The complete procedure provides a massive amount of synthetic oligonucleotide of an extremely high quality.

**Figure 2:**
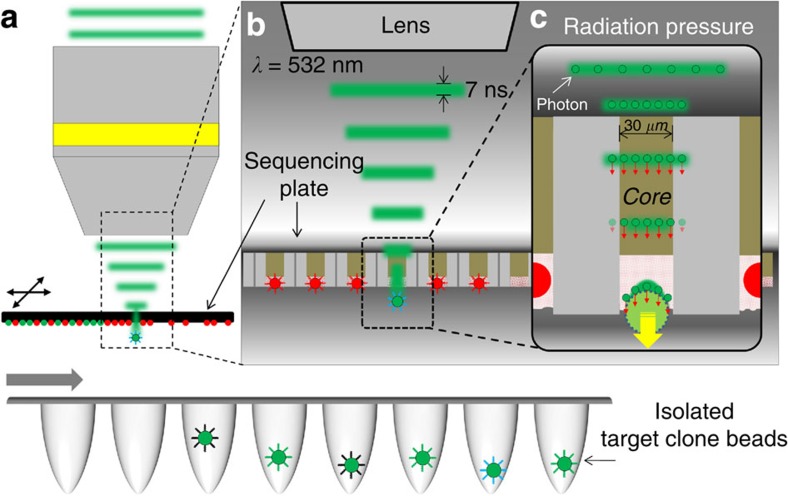
Schematic diagram of our focused pulse laser radiation pressure-driven non-contact target bead sniper system. (**a**) A motorized stage moves the sequencing plate and locates the target clone bead to the focusing spot of the pulse laser based on the real-world location information from our diffusion-like local mapping algorithm. Target clone beads were isolated into a PCR tube to directly utilize sequence-verified oligonucleotides on the bead surface. (**b**) The selectively etched fibre bundle structure of the 454 NGS substrate is well suited for optical releasing. The etched core region partially isolates a single clone bead while the remnant core delivers serial optical signals of pyrosequencing to the CCD. We used biocompatible 532-nm visible light to illuminate the side opposite to the bead-containing side and to couple the core region to carry the photon energy to the target bead penetrating through the sequencing plate. A nanosecond pulse effectively exerts a radiation force to target the bead without physical damage (longer pulse) or simple locoregional ablation (shorter pulse). (**c**) Since the fibre only carries light in the core region and otherwise attenuates light, it also minimizes the horizontal and vertical positioning error. This approach allows robust clone bead targeting without expensive optical and mechanical instruments. A single pulse with energy of ~50 μJ applies 0.25 μN of radiation force for target bead retrieval.

We took advantage of the optically favourable substrate structure of the 454 Junior platforms. Selective etching of the fibre bundle not only serves as an isolation chamber for each bead, but also provides successive optical pyrosequencing information from the substrate, which is delivered to the CCD front. As depicted in Fig. 2b, we inversely delivered a harmless, low-energy (50 μJ per pulse) visible nanosecond pulse laser (532 nm; 7 ns) to couple the laser pulses to the remnant core of the target well from the back side of the substrate. The remnant fibre core guides the light pulse that pushes the target clone bead with a radiation pressure (0.25 μΝ, 1.67 fNs impulse; Supplementary Note 4). The retrieved molecular clones are collected in a 96-well plate for subsequent amplification (Fig. 2a). Since the fibre only carries light in the core region and attenuates it elsewhere, the effects of positioning and fabrication errors are minimized for both horizontal and vertical coordinates of the substrate (Fig. 2c). This makes our system robust and eliminates the need for any expensive optical or mechanical instruments. Furthermore, the use of radiation or ablation (Supplementary Fig. 2)^24^,^25^,^26^ forces to drive the separation via focused light enables us to target clonal features with a very small size up to the diffraction limit (~1 μm) including beads, micro-circuits and even small debris of the substrate itself (Supplementary Figs 5 and 10), therefore most second-generation sequencing platforms with higher capacity are potentially available for the Sniper Cloning technique irrespective of whether they involve microstructure (GS series, Roche 454 Life Sciences; Iontorrent, Life Technologies) or directly attach DNA clusters on the surface (MiSeq, NextSeq, HiSeq; Illumina).

### Mapping algorithm for tracking clone features

**Figure 3:**
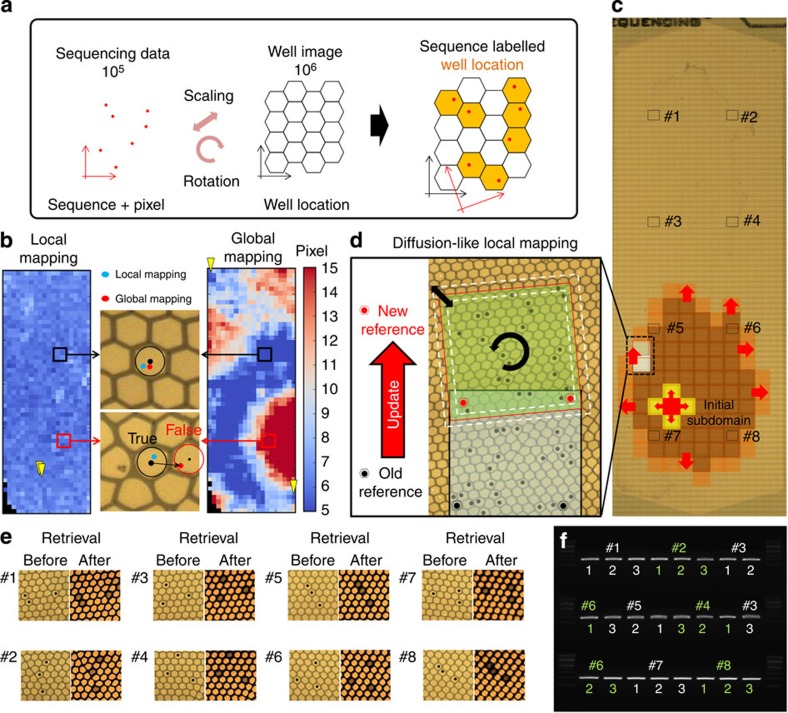
Diffusion-like local mapping algorithm for detecting target bead with real-world location on a chip. (**a**) Schematic diagram of our mapping algorithm. The 454 Junior normally offers ~10^5^ sequences with accordant pixel positions of CCD. From two arbitrary reference points, the corresponding sequence-labelled well location can be determined by adjusting for the scale and rotational angle of the sequencing pixel domain and overlap. (**b**) However, due to random and non-linear distortion of the sequencer’s imaging system, one-step global transformation leads to locational error. Approximately 20% of the pixels are mapped in a false position that is not distinguishable, severely reducing the reliability of all of the location data (Supplementary Fig. 22). Yellow flags indicate the reference points of each mapping calculation. A colour bar shows the pixel-wise distance between the mapped pixels and accordant well centre. The threshold value is ~13.5 pixels. (**c**) We reduced the effect of imaging distortion to a negligible level by localizing the region of interest. The whole-chip area was divided into 300 subdomains with a slight overlap. (**d**) One subdomain completes the location mapping by supplying two new reference points to the adjacent subdomain. Local mapping propagates from the initial matched subdomains throughout the whole chip. (**e**) Twenty-four beads were retrieved from eight evenly distributed regions to verify the local mapping algorithm. The left side of the figure describes the target well location on the stitched chip image and the right side of the figure shows the correct retrieval results. (**f**) Retrieved beads were amplified and identified with the Sanger method, showing matched results for each of the reference sequences.

The true mapping position of the target clone beads on the sequencing substrate can be found by overlapping the pixel map from NGS data with a ‘well centre’ position map of the stitched whole-chip image (Fig. 3a). However, due to the random and non-linear distortion^27^ of the sequencer’s imaging system, it is difficult to recover the precise location of each sequence-verified clonal beads throughout the whole chip by simple linear transformation of the error-prone pixel values (Fig. 3b and Supplementary Figs 11–13). The only way to eliminate the positional error induced by physical distortion is to localize the region of interest that has an acceptable amount of distortion. Our self-designed ‘diffusion-like local mapping algorithm’, an imaging error reduction algorithm for arbitrary non-linear distortion of any NGS platform (Supplementary Fig. 21), divides the whole chip area by 300 semi-linear subdomains with a slight overlap (Fig. 3c). Then, the mapping calculations between pixels and the corresponding well locations are diffused throughout the whole chip from one initial subdomain containing two Sanger-verified reference beads by adjusting the scale and rotational angle of the pixel domain. Adjacent subdomains are consecutively mapped according to two new reference points in the overlapping region, which are determined by the mapping results of the previous subdomain (Fig. 3d). Finally, 10^5^ sequence-labeled well locations are determined out of a total of 10^6^ wells from the stitched whole-chip image.

To determine the feasibility of our mapping algorithm, we retrieved 24 target beads from eight evenly distributed regions (Fig. 3c). A motorized stage moved the sequencing plate to the pulse laser focal point within the margin of positional error and retrieved the target beads resulting in an empty well, as seen on the right side of Fig. 3e. All retrieved beads were amplified and verified by Sanger sequencing (Fig. 3f). We further verified our Sniper Cloning approach with a pool containing a knockdown recombinant library by generating 1,380 short hairpin RNAs (shRNAs) that target 147 human protein-coding genes (aminoacyl-tRNA synthetases; Supplementary Data 1 and Supplementary Figs 14–16). The sequences were synthesized by a DNA microarray. After library amplification with random barcode and 454 adaptor primers, we used NGS to identify 1,338 (97%) perfectly matched sequences out of 77,940 clones, and retrieved 1,108 beads using our pulse laser system. To acquire a serviceable amount of product, additional amplification was conducted. Gel images indicate 1,035 (92.5%) clear bands and 83 unclear or undetectable bands. Interestingly, further NGS-derived sequence verification of the amplified products from 1,108 beads returned 1,060 perfect parts (95.67%), including 58 of the 83 samples with unclear bands with relatively low coverage (Supplementary Fig. 23). We believe that the 4.3% loss originated from sequencing error, bead damage during the sequencing run or storage, imperfect PCR conditions, or the retrieval process, and therefore consensus sequencing (barcode tagging) and replicated retrieval (one sequence for at least two beads) would reduce the loss rate to almost zero.

### ‘Sniper Cloning’ over conventional *in vivo* cloning

The effectiveness of the ‘Sniper Cloning’ approach becomes more evident with a simple mathematical comparison (Supplementary Note 1 and 2) of the probability of retrieving desired sequences when compared with the conventional approach. Given a complex colony library of ‘*n*’ kinds of unique sequences with uniform distribution, the probability ‘*P*’ that a given unique sequence is selected in a collection of *N* pickings is as follows^11^:


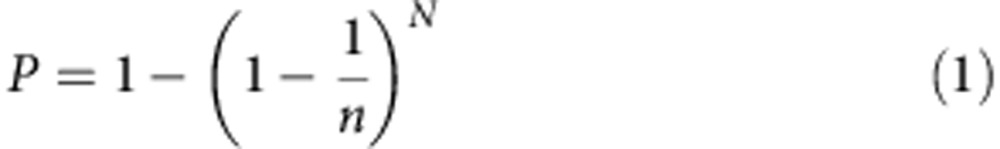


Thus, the total probability function to recover all ‘*n*’ contents out of a complex colony library is *P*^*n*^. In the case of *n*=1,000, numerical simulation indicates that 10,004 rounds of random colony picking is needed to recover 95.6% of the content. Moreover, the complex mixtures synthesized by DNA microarray followed by library amplification generally suffer from synthesis error and amplification bias^28^. On a count of 10 × bias and 50% synthesis error, which is a very conservative assumption, the necessary random picking and individual sequencing work exponentially increases by orders of magnitude (60,394 times), corresponding to ~6,000 plates, whereas our approach requires just one 454 Junior sequencing run with a minimal amount of automated separation work.

Even if the sequencing plate has only one perfect target within the entire population, it can be used without any additional work than species with a large population. We prepared a more complex DNA pool containing 10,634 different sequences of human protein-coding gene targeting shRNAs (Supplementary Data 2). Even though NGS results indicated relatively poor quality of the microarray DNA pool and the effect of library amplification bias, we identified 5,188 (48.8%) perfect targets. Further, 99% of the perfect targets have fewer than 10 copies and 48% have only 1 copy each (Supplementary Fig. 24). We successfully separated 5,188 beads from the sequencing plate within a week; 2 days of microarray DNA pool synthesis, 2 days of library amplification and parallel identification, 1 day for Sanger-derived reference bead determination and 2 days for mapping and retrieval (Supplementary Figs 17–20). Further optimization of microarray synthesis and library amplification to increase the quality of the amplified pool could lead to more extensive population coverage. Also, the throughput can be increased at least 10-fold if applied to the 454 GS-FLX platform.

**Figure 4:**
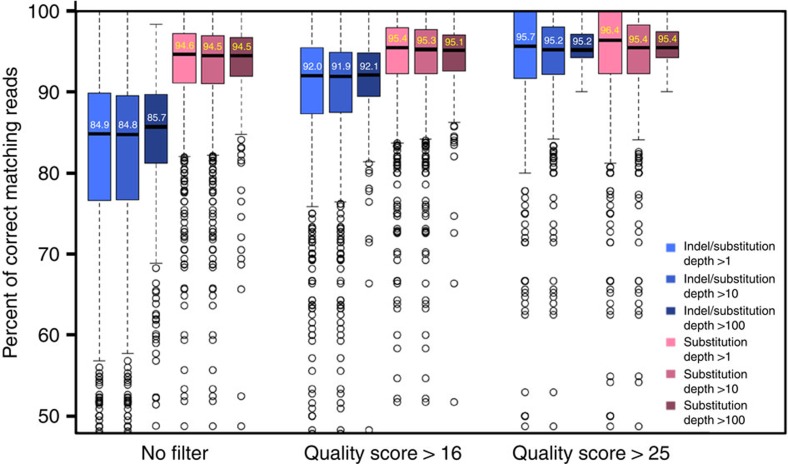
NGS-based quality analysis of retrieved DNA. The box plot presents the distribution of correct reads in the verification NGS run of retrieved bead. A total of 96,484 molecules (1,010 kinds) were arranged with respect to the quality score and depth. Blue boxes show the correct read distribution considering both indel and substitution mismatches, whereas red boxes take only substitution into account. The median percentage values of the blue boxes in group 1 were in the mid-eighties, which is almost 10% less than those of the red boxes in the same group. However, these values gradually increased in the group of higher quality score reads, reaching the almost constant red box values for group 3 (95.7%). These results indicate that the majority of indel mismatches are sequencing errors that originated from error-prone homopolymer pyrosequencing. Therefore, we can say that the percentage of correct DNAs retrieved is >96%.

To evaluate the quality of the separated DNA, we analysed 454 sequencing data of 1,010 retrieved bead amplicons. Figure 4 describes the proportion of correct sequences in each sub-pool of different quality score reads. Red boxes show the proportion of perfect reads, considering only substitutional error, whereas blue boxes take both substitutional and indel error into account. As the sequencing quality score increases, the median values of the blue boxes rapidly approach those of the red boxes. This means that the majority of indel error reads come from NGS sequencing errors and thereby we can estimate the quality of the retrieved DNA to be at least 96% with an estimated error rate of 1 in 2,367 bp. Considering an error rate of initial pool oligonucleotides of 1 in 70 bp and a 22.3% median accuracy, Sniper Cloning enhances the quality by ~34-fold (Supplementary Note 5).

To the best of our knowledge, it is very difficult to supply a massive amount of synthetic oligonucleotides of extremely high standard, except through conventional cloning and random pick-and-place followed by individual identification (Supplementary Note 3). Although the linear form of synthetic DNA is used in the majority of cases, a considerable portion of end users are still demanding circular or vector formation of DNA^29^. The very low error rate of our products allows a direct clone-and-use strategy that eliminates the subsequent selection and identification process (Supplementary Figs 25 and 26). We sequenced 55 shRNA inserts and found that 96% (52 samples) were perfectly correct clones, consistent with the previous NGS analysis. The remaining three inserts had single base-pair mismatches (Supplementary Fig. 27 and Supplementary Method 2). Although the insert to vector ratio varied from 50 to 90%, enough to be used as a gene manipulator can be attained (Supplementary Fig. 28).

## Discussion

In summary, our Sniper Cloning approach provides massive amounts of ultra-high quality synthetic oligonucleotides. A custom pulse laser retrieval system enables non-contact contamination-free high-throughput separation of accurate sequences from the sequencing plate with precise position data obtained from a diffusion-like local mapping algorithm. The serial process consists of parallel synthesis, massively parallel identification and high-throughput separation, which dramatically reduces the cost, time and labour by eliminating the randomness of conventional cloning. The development of optomechanical separation and imaging error reduction techniques bridges the gap between next-generation ‘reading’ and ‘writing’. We believe that our ‘Sniper Cloning’ platform directly utilizes the power of NGS reading to enhance DNA writing, serving an essential role in protein engineering, functional genomics, synthetic genomics and synthetic biology in general.

## Methods

### Amplification of a microarray-derived oligonucleotide pool

A DNA pool library was synthesized with a CustomArray B3 Synthesizer using a 90 K array chip. We used KAPA library amplification kit (2 × KAPA HiFi HotStart ReadyMix, KAPA Biosystems, Boston, MA, USA) to minimize the amplification bias. Amplification of the pool library was performed with custom designed primers (Bioneer, Daejeon, Korea) of 26-mer universal sequences with overhangs of 454-A and 454-B for Roche/454 sequencing. PCR conditions were 10 μl 2 × KAPA HiFi HotStart ReadyMix and 1 μl (20 μM) each primer with cycling parameters of initial denaturation at 98 °C for 3 min, followed by 20 cycles of 98 °C for 30 s, 55 °C for 30 s, 72 °C for 30 s, with a final elongation at 72 °C for 5 min. After amplification, we extracted PCR products of the appropriate length by agarose gel electrophoresis for emulsion PCR.

### Sequence-verified clone generation (454 GS Junior)

The initial preparation process was performed with the amplified double stranded DNA of the microarray-derived oligonucleotide pool according to the protocols of GS Junior from Roche 454 Life Sciences. Sequencing was also conducted according to the protocols except for the final washing step. We aborted the sequencing process immediately before the bleach solution washing step to prevent DNA damage on the surface of microbead. Instead of the final washing step, we applied a maintenance wash kit with a dummy chip at every run.

### Optomechanical retrieval setup

The system included a Q-Switched Nd:Yag laser system (Minilite, Continuum, 28 mJ at 1064, nm, 12 mJ at 532 nm, 4 mJ at 355 nm, 2 mJ at 266 nm, repetition rate: 1–15 Hz), true-colour charge-coupled device (CCD) cameras (Guppy PRO F-146C, ALLIED) and two motorized stages, a top one (SCAN IM120 × 100, MärzhäuserWetzlar) for the sequencing plate and a bottom one (SCAN 100 × 100, MärzhäuserWetzlar) for the PCR plate, which were controlled by a personal computer with self-made Labview software. The upper part of the system, a commercial inverted microscope (IX71, Olympus) with a × 10 objective lens and a motorized stage, was hung upside down such that the direction of the radiation force was identical to that of gravity. We constructed the whole system, except for the personal computer and pulse laser power supply, on an anti-vibrational optical table (Supplementary Method 1).

### Sequence verification of the retrieved DNA (bead)

Each of the retrieved beads was individually re-amplified for sequence verification. PCR conditions were 10 μl 2 × p.f.u. polymerase pre-mix (Solgent, Daejeon, Korea) and 1 μl (20 μM) each primer with cycling conditions of initial denaturation at 95 °C for 3 min followed by 25 cycles of 95 °C for 30 s, 60 °C for 30 s, 72 °C for 30 s and final elongation at 72 °C for 5 min. All PCR products were analysed by both agarose gel electrophoresis and the NGS platform (Roche/454 GS Junior+).

### Image processing and data analysis

Image processing including stitching, centre recognition, rotation and mapping calculation were mainly performed by Python and Matlab script with the help of built-in functions. Oligonucleotide pool design and perfect matching sequence selection were conducted by a custom Matlab script.

## Author contributions

HW.L, HK.K, DH.B and SH.K proposed the concept of the work and designed the experiment. HW.L, HK.K and SS.K performed the optical experiments and analysis. SS.K worked on the computational algorithm for image processing and location mapping. TH.R and HB.K performed biological experiments.

## Additional information

**How to cite this article**: Lee, H. *et al*. A high-throughput optomechanical retrieval method for sequence-verified clonal DNA from the NGS platform. *Nat. Commun.* 6:6073 doi: 10.1038/ncomms7073 (2015).

**Accession codes:** Sequence data for a pool containing a knockdown recombinant library by generating 1,380 shRNAs that target 147 human protein-coding genes have been deposited in GenBank/EMBL/DDBJ nucleotide core database under the accession code SRP050235.

## Supplementary Material

Supplementary Figures, Supplementary Notes, Supplementary Methods and Supplementary References. Supplementary Figures 1-28, Supplementary Notes 1-5, Supplementary Methods and Supplementary References

Supplementary Data 1 1st pool sequence (sequence information attached with excel file: Supplementary Data 1. 1st pool sequence). To confirm system feasibility, we constructed a shRNA library pool of 1,528 (1,338 after repetition removal) clones targeting 147 human protein-coding genes (aminoacyl-tRNAsynthetases) using a DNA microarray synthesizer.

Supplementary Data 2 2nd pool sequence (sequence information attached with excel file: Supplementary Data 2. 2nd pool sequence). From a library with a total of 330,461 shRNAs in the RNAi consortium (TRC, Broad institute), we choose 19,061 shRNAs for protein-coding gene knockdown (http://www.genenames.org/cgi-bin/hgnc_stats). After filtering out non-appropriate sequences for pLKO1 vector cloning, we generated a library containing 10,634sequences. The structure and amplification conditions of the second library were the same as for Supplementary Data 1.

Supplementary Movie 1 Demonstration of opto-mechanical retrieval of clonal DNA (beads) with the pulse laser system. The throughput reached up to 2 beads per second as shown in the movie.
