# Supplementary material for: A high-throughput optomechanical retrieval method for sequence-verified clonal DNA from the NGS platform
Source: Nat Commun. 2015 Feb 2;6:6073. doi: 10.1038/ncomms7073 (PMC4327316; doi:10.1038/ncomms7073)
Supplement: Supplementary Figures, Supplementary Notes, Supplementary Methods and Supplementary References. — Supplementary Figures 1-28, Supplementary Notes 1-5, Supplementary Methods and Supplementary References [file ncomms7073-s1.pdf]

Supplementary Figures

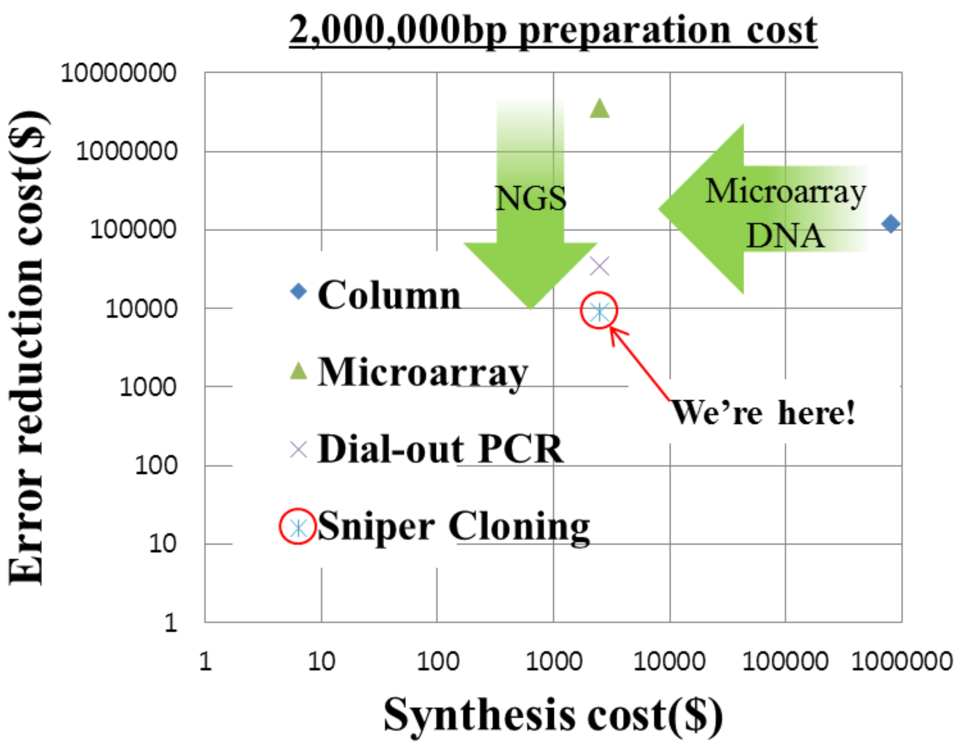

Supplementary Figure 1. Cost comparison graph. (Conventional vs. 'Sniper Cloning') – Cost references at Supplementary Note 1.

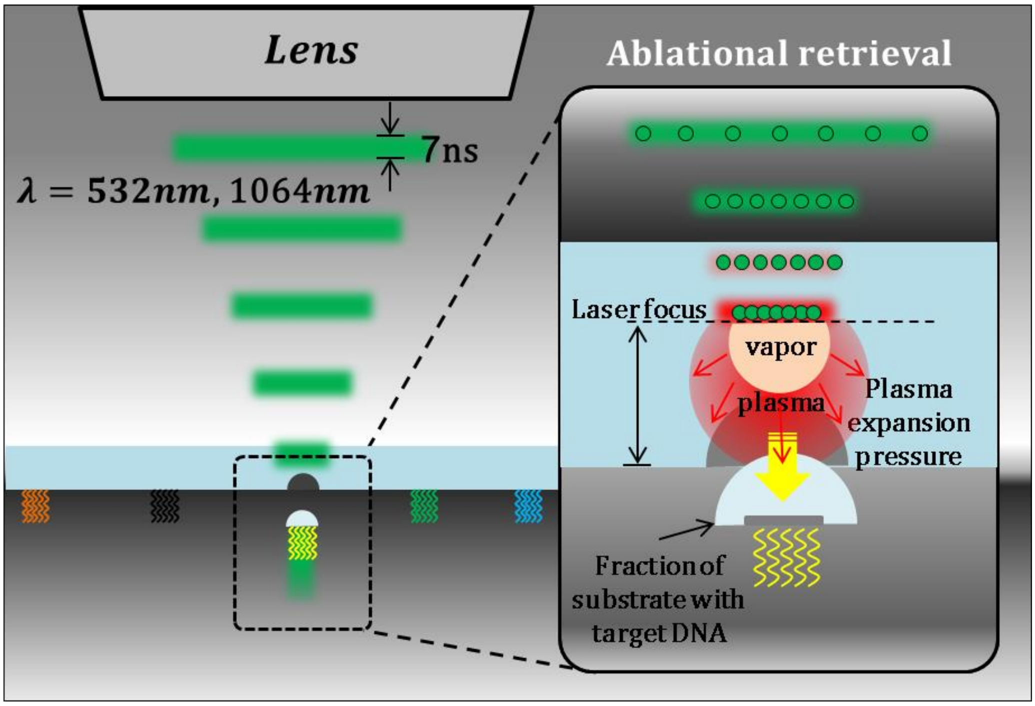

Supplementary Figure 2. A schematic diagram of direct ablational retrieval. To retrieve sequence verified DNA from the substrate of Illumina sequencer, we use plasma development pressure caused by pulse laser ablation of substrate. As shown in the figure, we intentionally focused laser pulse slightly above (inside) the surface of substrate. High energy of focused laser pulse turns small volume of substrate into plasma status. The force of those expansion pushes target substrate region together with target DNA sequence to PCR tube.

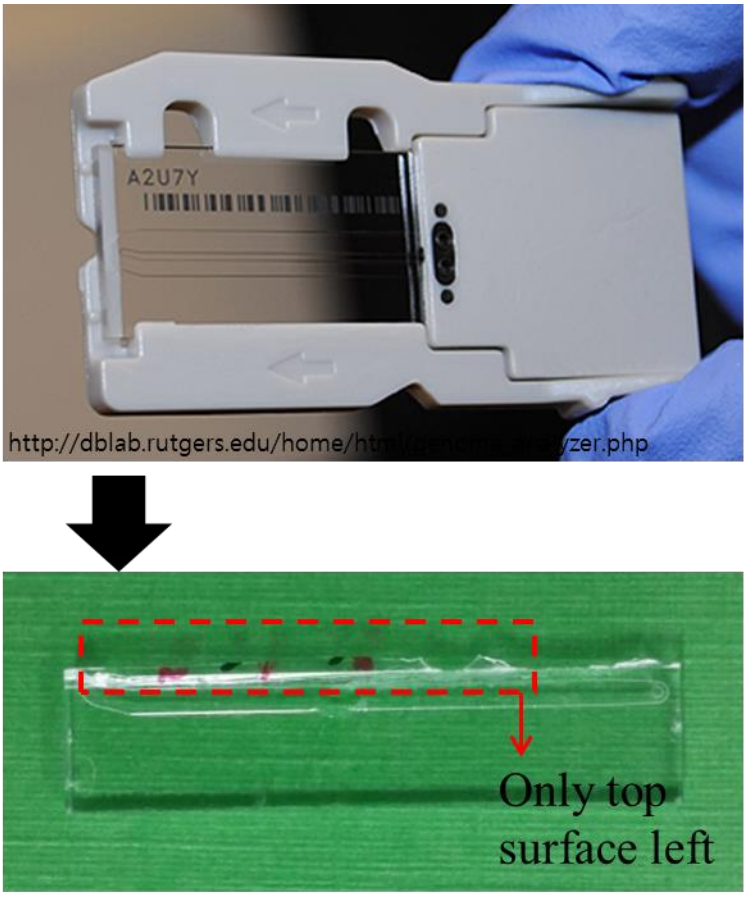

Supplementary Figure 3. Disassembly of MiSeq flow cell for direct ablational retrieval. Figure shows a broken piece of Illumina MiSeq plate for ablational retrieval (We are now trying to separate flow cell of sequencing substrate using laser cutter for more precise disassemble). Current hardware and software upgrade of Illumina sequencer offers sequencing information of DNA clusters attached on both top and bottom surface. After sequencing, DNA clusters and their fluorescence signals were conserved due to the modified protocol of skipping last bleaching (washing) step of Illumina sequencer.

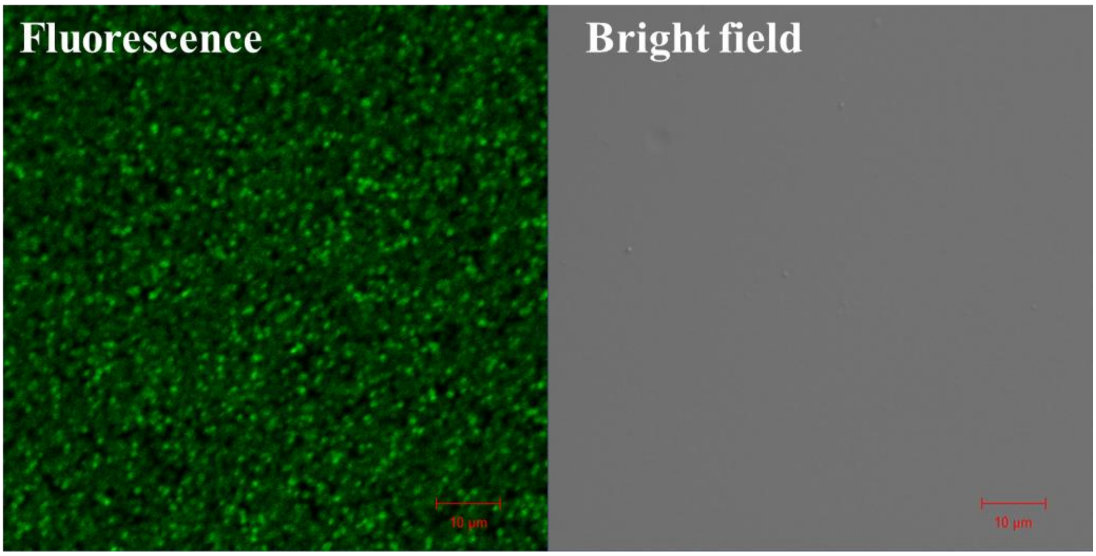

Supplementary Figure 4. Confocal image of fluorescently labeled DNA clusters on top surface of MiSeq flowcell. Excitation laser (488nm), Filters (496-606nm), Objective (20×, with 5× digital zoom)

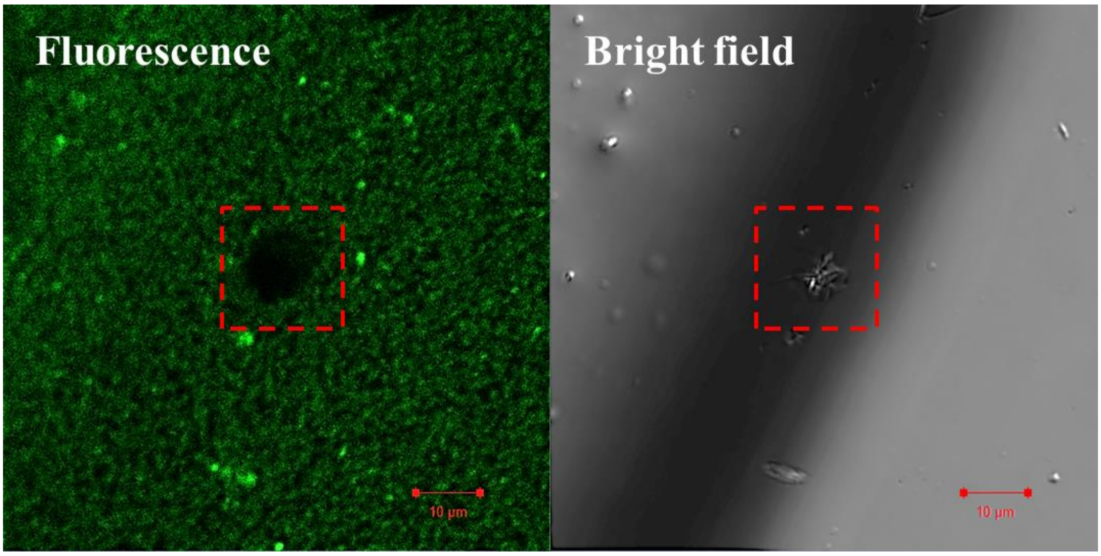

Supplementary Figure 5. Fluorescence image of cluster surface after ablational retrieval. Figure shows the fluorescent image and bright field image of substrate after ablational direct retrieval process. Thanks to the characteristics of nanosecond pulse laser, the minimal retrieval size of this method is limited around 10 μ m diameter.

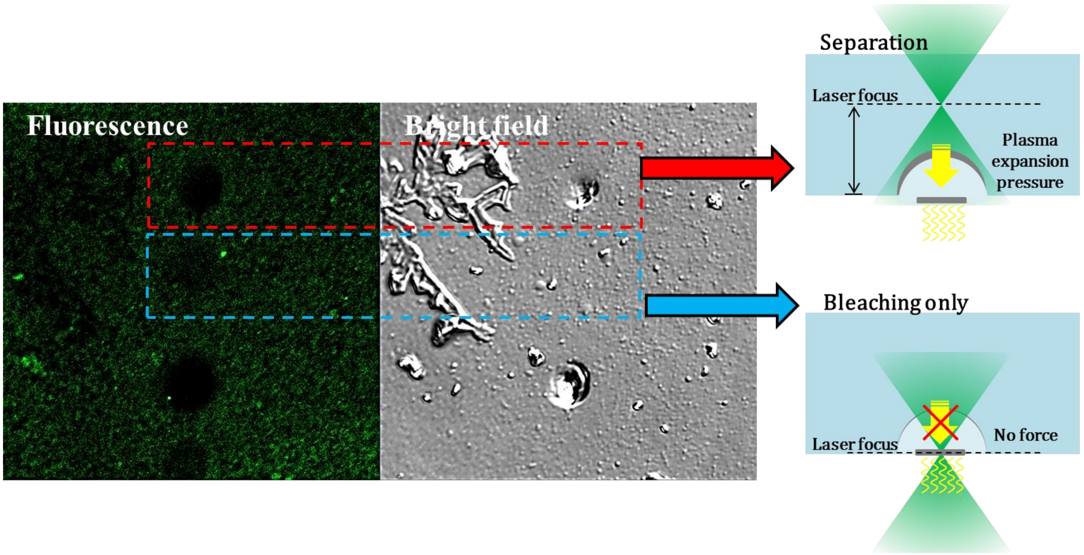

Supplementary Figure 6. The laser focus dependency of ablational retrieval. Plasma expansion pressure only occurs when the laser focal spot stays inside the substrate.

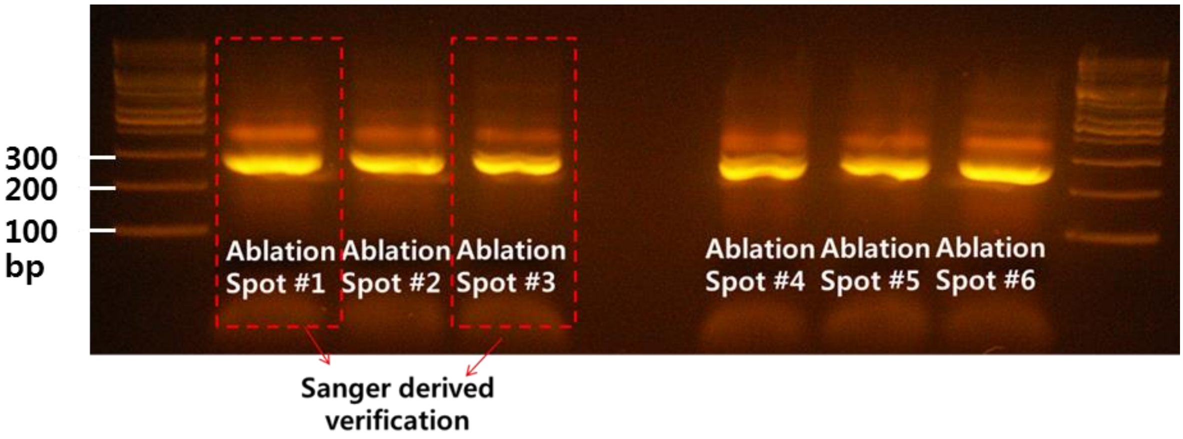

Supplementary Figure 7. Gel image of amplified products from ablation debris. We amplified the debris from the ablation process with universal primer. Gel image clearly shows the amplified product. This product, again, is sequence-verified by TOPO cloning followed by conventional Sanger derived sequencing.

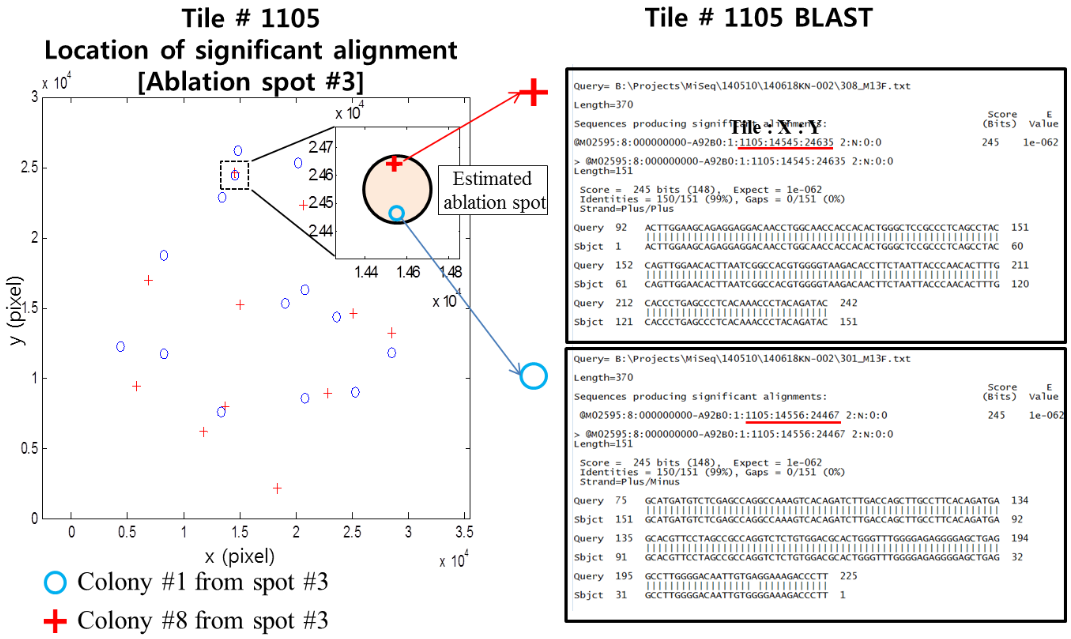

Supplementary Figure 8. (Left) Pixel locations of significant alignment sequences. (Right) BLAST matching results. Sanger sequencing results of colony #1 and #8 from ablation spot #3 have significant alignment sequences indicating blue circle and red plus each in the pixel location graph. Two pixel locations with high matching score for each colony sequence were found in close proximity that are expected to be retrieved from same ablation spot #3. Since the size of the ablation spot generated by nanosecond pulse is larger than those of average cluster (1 μ m), more than one species are observed by the Sanger result. Two ablation spots are physically far apart to locate different tiles (MiSeq flow cell imaging consists of 14 tiles each-top and bottom surface- totally 28 tiles). We conducted BLAST with Sanger results as queries and Illumina data as a subject. For each ablation spots, Sanger results of two colonies, which had significant alignment sequences within very adjacent local pixel numbers (within 100pixels) as shown in Figure. According to the report, 1um size single cluster occupies around 10 pixels. Then, 100pixel local proximity is reasonable result considering our ablation spot size (10 μ m). We're trying to optimize the optic system and pulse width (from nanosecond to picosecond) to reduce the size of the ablation spot for general use in bead less platform such as Illumina sequencer.

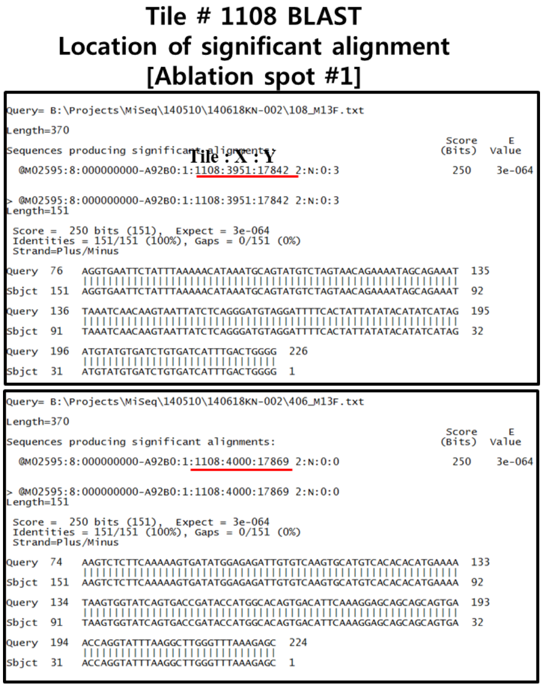

Supplementary Figure 9. BLAST matching results of colony #8 and #6-1 from ablation spot #1.

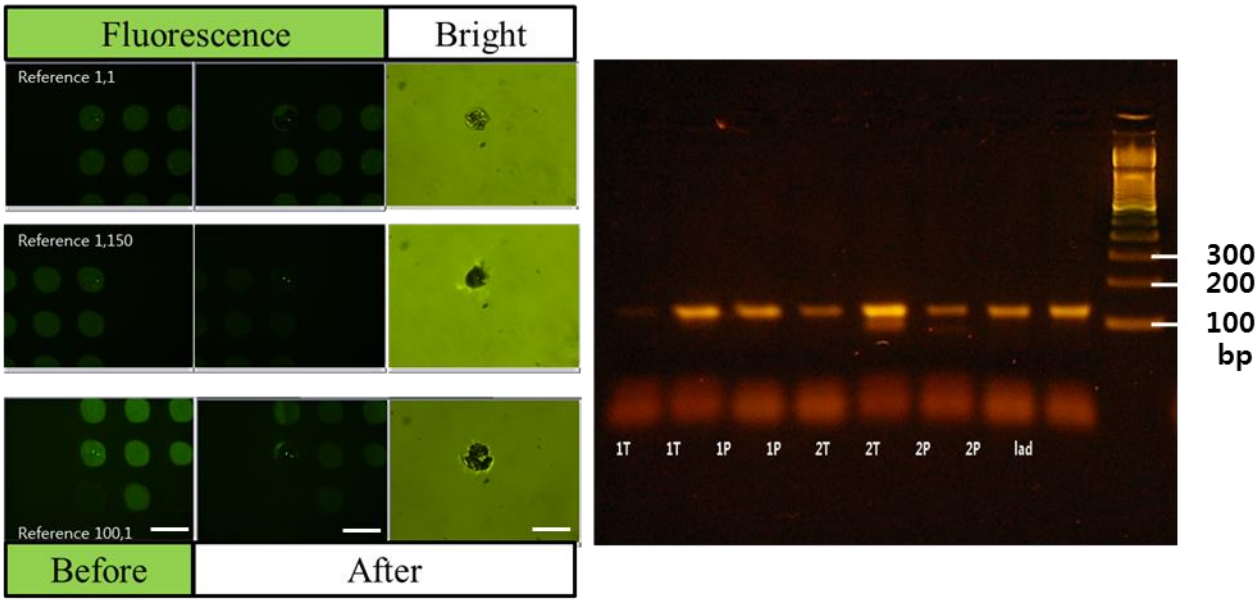

.

Supplementary Figure 10. (Left) Fluorescence image of DNA microarray (scale bar 50 μm). Three spots were targeted by direct ablational retrieval. (Right) Gel image of PCR product of debris from microarray. Since the size of the spot of DNA microarray is usually larger (30 μ m) than those of sequencing platform, this figure will help readers' understanding.

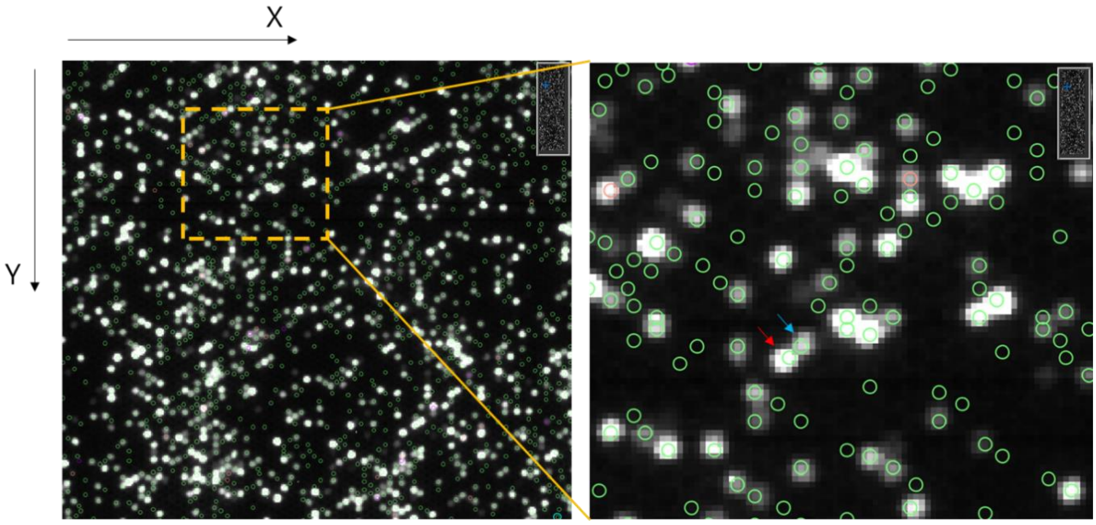

Supplementary Figure 11. Random assignment distortion of pixel data. 454 GS Junior acquires sequential optical signals that contain sequence and location information of each bead on the sequencing substrate through tapered optical fiber and CCD camera. Images captured with a tapered optical fiber show significant spatial distortion mainly because the spatial orientation of the fiber bundles is not identical at each end of the taper [1]. Also, for addressing signal location of each bead, the physical locations of the beads are mapped to CCD pixel addresses. In this process, quantization error occurs, as the physical location of a bead on a sequencing substrate has a continuous value while the CCD pixel has a digitized value. Therefore, the address of each sequence provided in the 454 sequencing raw data contains errors. Even if these errors are small, they generate critical mistakes during inverse mapping from the CCD pixel to a physical location for retrieving targeted beads. Figure shows the typical quantization errors. The image is captured by a built-in CCD camera in a 454 Junior sequencer. Colored circles represent locations of sequenced beads and one circle corresponds to one read in the raw data. Each circle has its unique positional information, which is the CCD pixel address designated in the raw data. Two arrows (red and blue) in the right side of the figure show a typical image of the adjacent beads, which have quantization errors in the positional information. The sequence shown by the red arrow has a bigger X and smaller Y position than its signal center. However, the sequence shown by the blue arrow has a smaller X and bigger Y position than its signal center. Most of beads have this kind of error in their positional information.

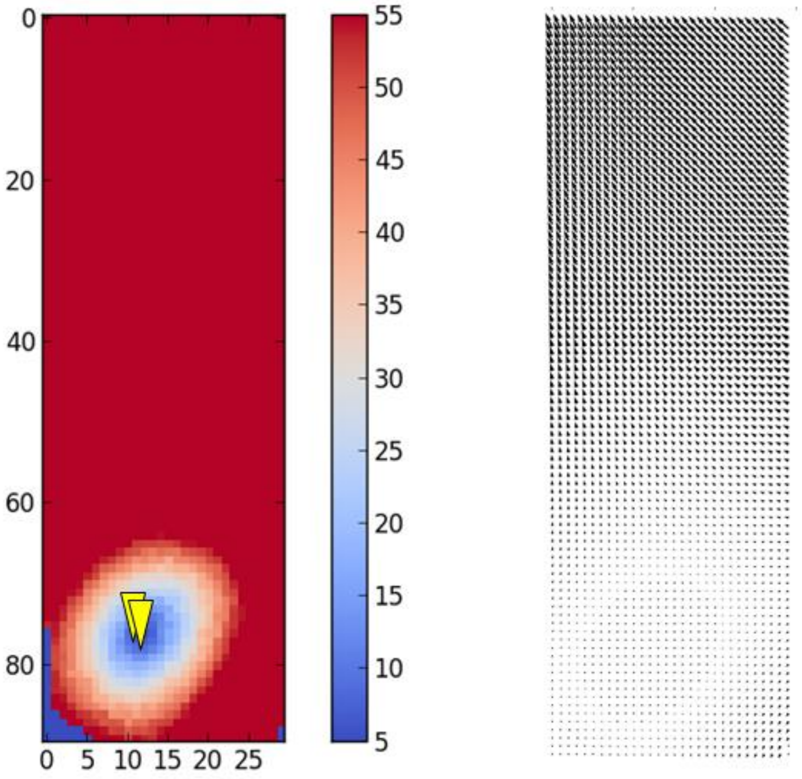

(A) (B)

Supplementary Figure 12. Distance between the well center and the mapped pixel deviation value map of the close reference points. Yellow flags indicate the location of the two reference points. The unit of the color bar is pixel (threshold = 13.5pixel). (A) Color map of deviation value. (B) Deviation vector map. In the case of close reference points, errors concentrically propagate through whole chip from the references. We think this mainly comes from the quantization error of the imaging system.

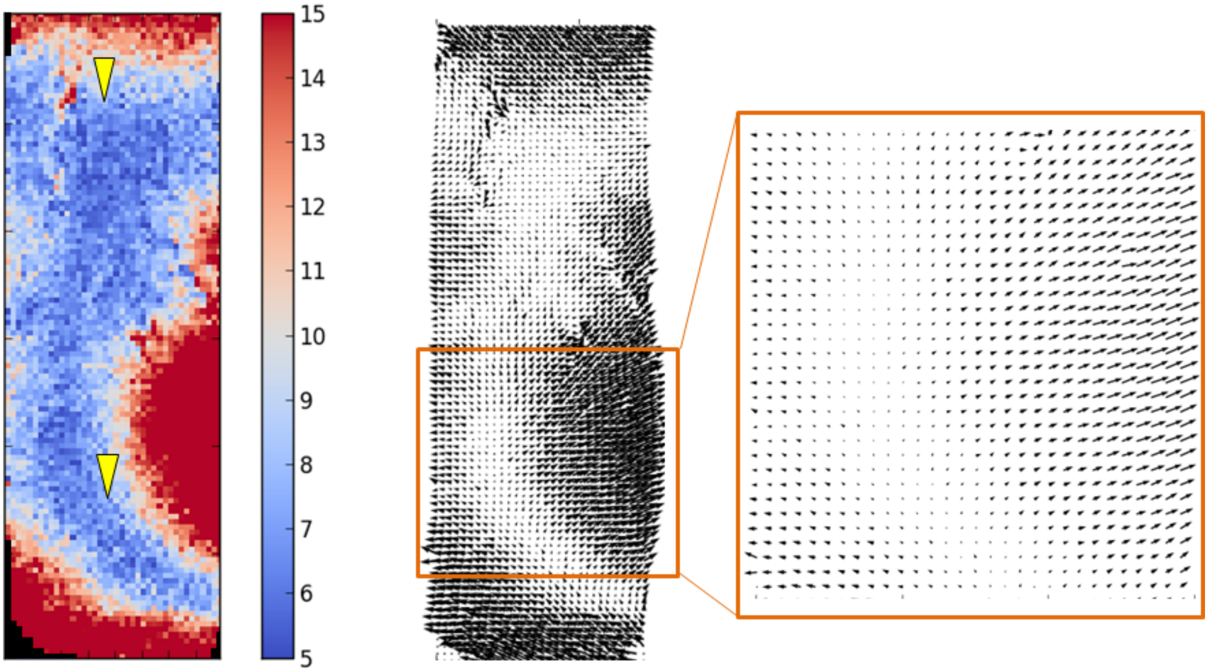

(A) (B) (C)

Supplementary Figure 13. Deviation value map of sparse reference points. (A) Color map of deviation value. (B) Deviation vector map and its magnification view (C). In the case of sparse reference points, the tapered fiber nonlinear distortion error is significantly shown over the quantization error in the specific region of the chip.

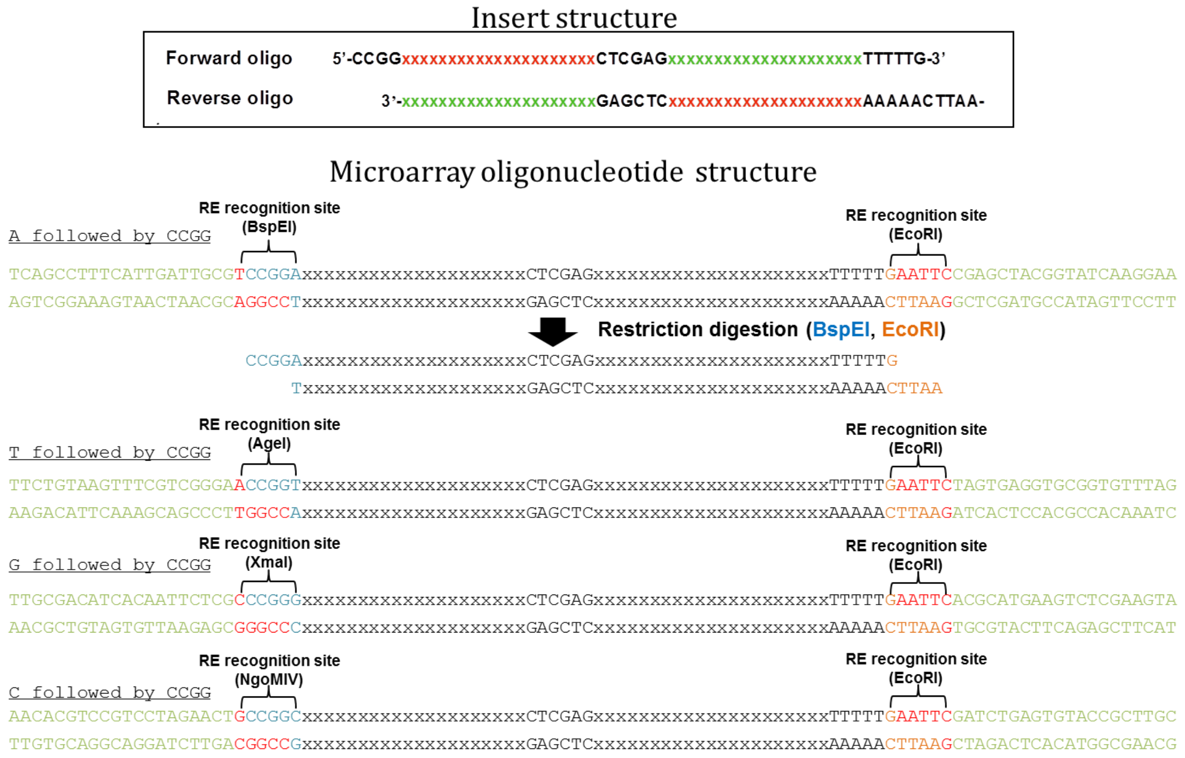

Supplementary Figure 14. shRNA sequence structure for microarray synthesis. The internal structures of the target shRNA sequences are depicted as figure.The target sequence structure consists of shRNA inserts with a primer region, and restriction recognition sites at both ends. To generate identical overhang sequences for cloning, we designed four types of structures according to the insert sequence. BspEI, AgeI, XmaI and NgoMIV recognition sites are designed between the front primer region and insert sequence and an EcoRI recognition site is located at the rear. We attached a 454 adaptor for high-throughput sequencing by amplifying the pool library with primers that have a 454 adaptor overhang and 10mer random barcodes.

shRNA_A_454_For	CCA TCT CAT CCC TGC GTG TCT CCG ACT CAG NNN NNN NNN NTC AGC CTT TCA TTG ATT GCG
shRNA_A_454_Rev	CCT ATC CCC TGT GTG CCT TGG CAG TCT CAG NNN NNN NNN NTT CCT TGA TAC CGT AGC TCG
shRNA_T_454_For	CCA TCT CAT CCC TGC GTG TCT CCG ACT CAG NNN NNN NNN NTT CTG TAA GTT TCG TCG GGA
shRNA_T_454_Rev	CCT ATC CCC TGT GTG CCT TGG CAG TCT CAG NNN NNN NNN NCT AAA CAC CGC ACC TCA CTA
shRNA_G_454_For	CCA TCT CAT CCC TGC GTG TCT CCG ACT CAG NNN NNN NNN NTT GCG ACA TCA CAA TTC TCG
shRNA_G_454_Rev	CCT ATC CCC TGT GTG CCT TGG CAG TCT CAG NNN NNN NNN NTA CTT CGA GAC TTC ATG CGT
shRNA_C_454_For	CCA TCT CAT CCC TGC GTG TCT CCG ACT CAG NNN NNN NNN NAA CAC GTC CGT CCT AGA ACT
shRNA_C_454_Rev	CCT ATC CCC TGT GTG CCT TGG CAG TCT CAG NNN NNN NNN NGC AAG CGG TAC ACT CAG ATC

Supplementary Figure 15. Primer sequence for library amplification. The primer sequence consisted of a 454 adaptor, 10mer barcode and primer region.

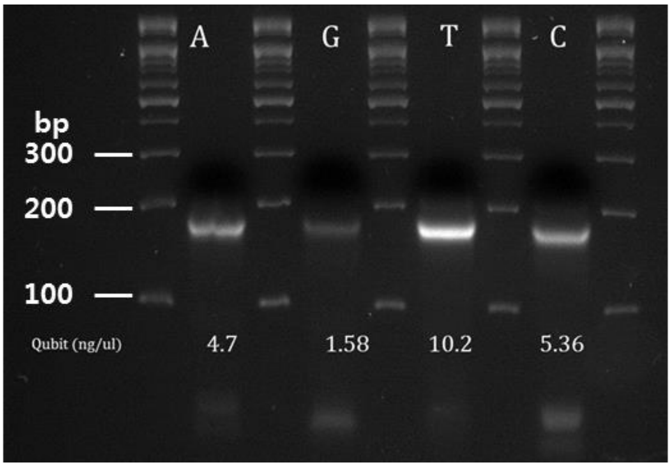

KAPA library amplification premix kit	10	µl
Forward primer	1 (20uM)	µl
Reverse primer	1	
Template	3.4(A)<br>0.85(G)<br>3(T)<br>0.8(C)	µl
water	4.6	µl
cycle	**19+1**	Last 1 cycle with 1ul primer addition
98(180) , [98(30), **55(30),** 72(60)]x18, 72(300),4 forever		

Supplementary Figure 16. Gel image and conditions for library amplification. We used library amplification polymerase (KAPA HiFi Library Amplification Kit, KAPA) to reduce PCR bias. For each reaction, the templates were added at a ratio predetermined from the library design. One cycle of amplification was conducted with additional 1µl of forward and reverse primers.

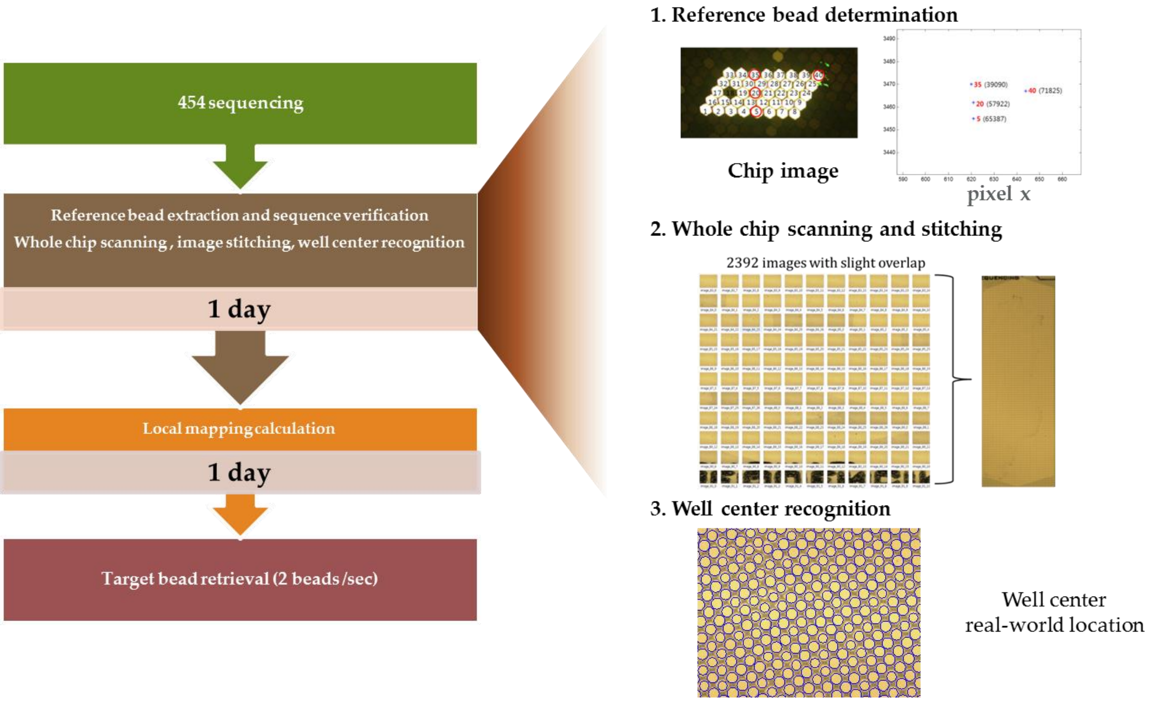

Supplementary Figure 17. Work flow of local mapping process. First, 454 sequencing is performed following standard protocol except that the run is aborted just before the bleaching solution injection. When sequencing is finished, the 454 picotiter plate (PTP) is placed in the retrieval system. Tens of sequencing beads are extracted by the system and physical location data of the beads are registered for post process. Each bead is PCR-amplified and sequenced

by Sanger method. Sanger-derived sequencing results are analyzed in the computer to find the CCD pixel information corresponding to each sequencing read. The pixel data are linked to physical location data for all analyzed beads and this linkage data is used for the initial conditions of the in-house diffusion-like local mapping algorithm. In parallel, an image of the 454 PTP is scanned by a computer. An optical microscope with a 10× lens is used for imaging. The whole chip image is divided into 2000~3000 small views and each view is saved with the appropriate index. Then, the collected images are stitched into one whole-chip image and the well center data in the whole-chip image are generated by an image recognition process. Linkage between bead positions of the CCD pixels from the 454 raw data and well center data from image processing is used as an initial starting point for whole-plate mapping. Mapping information through whole plate is constructed by utilizing an in-house diffusion-like local mapping algorithm that generates precise locational information in the 454 PTP for all sequences in the 454 raw data. This takes about 1 day to run on a personal computer. Finally, the mapping information is used for target bead retrieval. The 454 PTP is placed on the retrieval system and the PTP is automatically moved for the targeted bead to be extracted by laser radiation, which is a non-contact, high-throughput process.

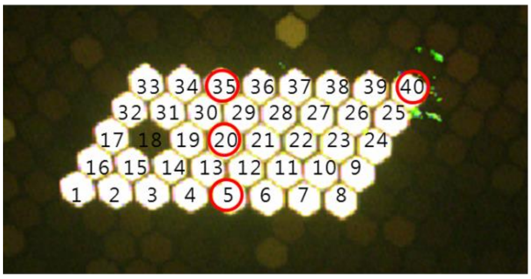

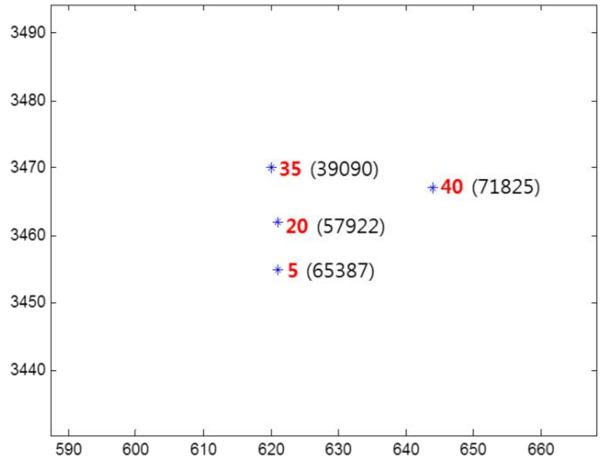

(A) (B)

Supplementary Figure 18. Determination of reference beads. To connect pixel information and real-world well location data, tens of beads were extracted and verified by capillary sequencing as shown in the figure above. Since only 10% of wells contained passed filter sequence beads, we retrieved 40 beads to get at least two reference points within a single sub-

domain range. (A) Transmission micrograph of reference bead retrieval region. (B) Retrieved beads were amplified and sequenced by Sanger method to find the counterpart in the 454 sequencing data. We found 4 perfectly matched sequences.

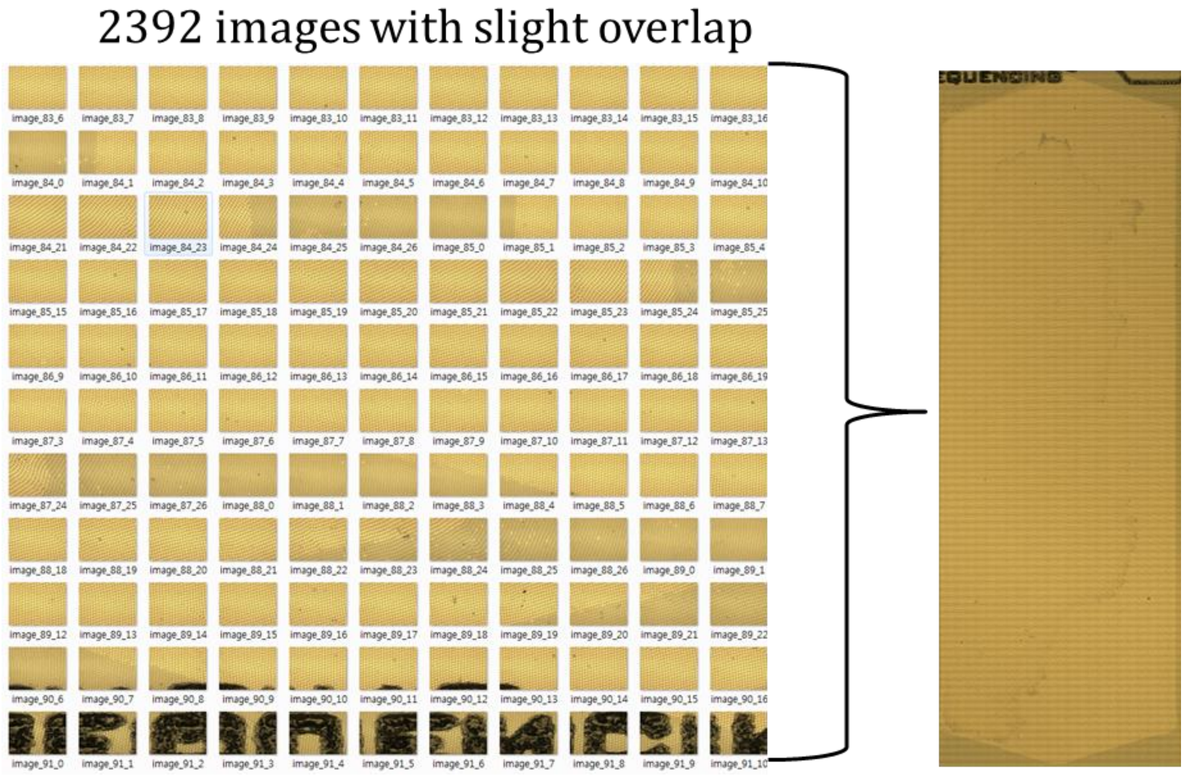

Supplementary Figure 19. Whole-chip image construction using a Python image stitching script. Images of a 454 Junior plate were scanned into 2000-3000 split images to get better resolution for post-image processing and well-recognition. The 454 PTP was moved by a motorized stage and images were acquired by CCD camera (Allied Vision Technologies). The whole process was automated under a Labview application. We combined 2392 images into one big image to generate a high-resolution whole-chip image. For optimal mapping process, an extremely accurate image stitch was required. Single-pixel mismatch can be fatal because accumulation of errors occurs through thousands of images and this generates distorted well positions in real-world location. This process was performed using a Python script.

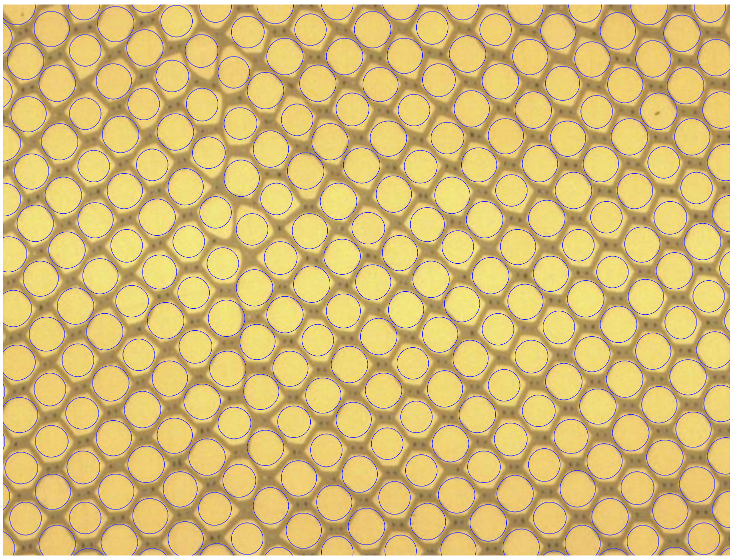

Supplementary Figure 20. Circle recognition to build relative well center location data. Relative location data of well centers throughout the whole-chip are generated from the stitched image. The MATLAB built-in function for circle recognition was used for this step.

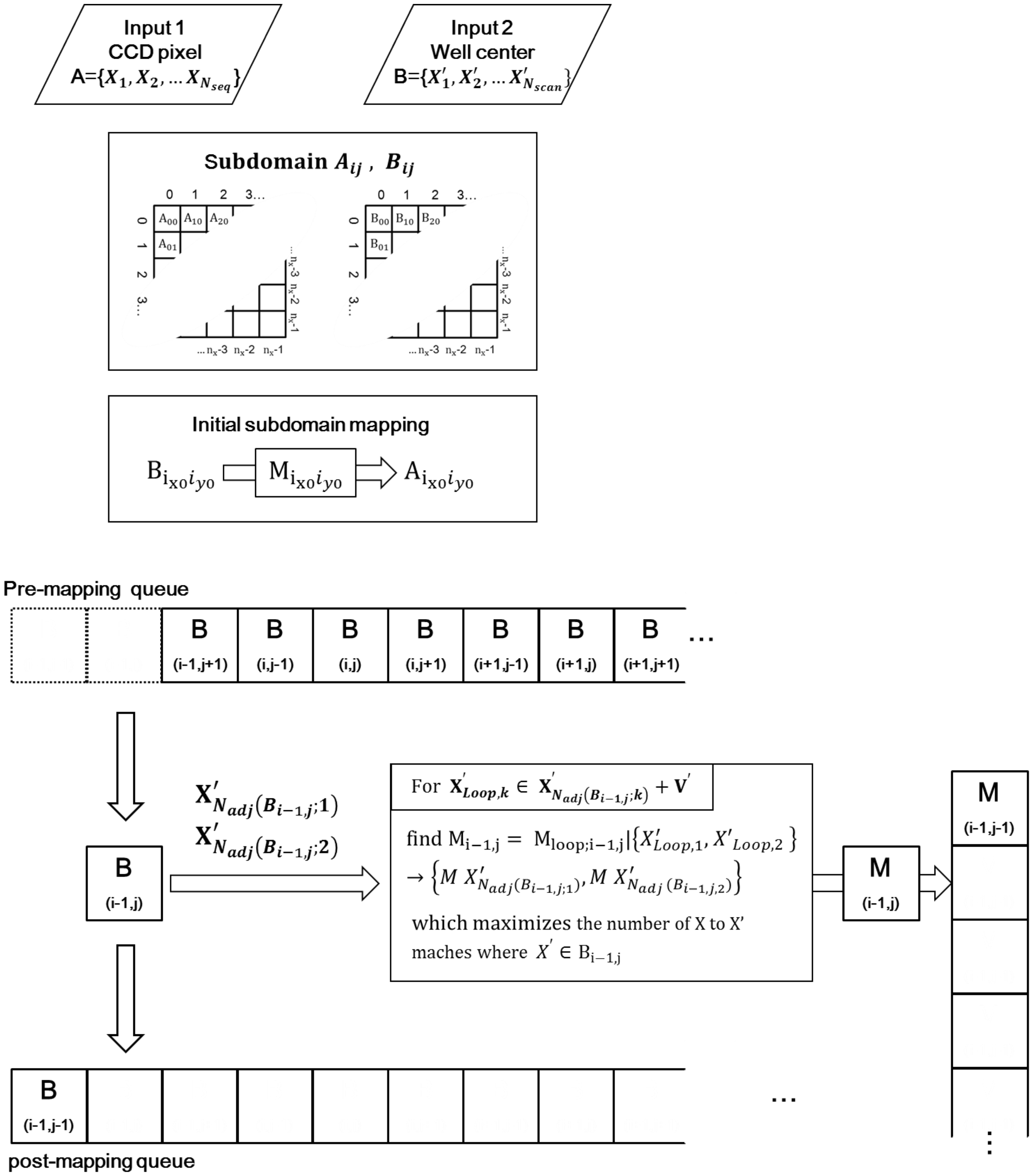

Supplementary Figure 21. Flow chart of diffusion-like local mapping algorithm. The algorithm used for mapping CCD pixels of the NGS raw data to well center data is represented in the figure. CCD pixels from 454 raw data and well centers from the stitched image are used as input. Each data set was divided into subdomains according to their positions. The reference points were extracted from the initial subdomain. Utilizing the linkage data of the reference points, we generated a matrix that transforms the well center to CCD pixel data. The subsequent mapping matrices were computed iteratively. After

calculating the mapping matrix, the subdomains having adjacent indices were put into the pre-mapping queue only if they were not in the post-mapping queue. The subdomain used to compute the mapping matrix was placed in the post-mapping queue. The first element in the pre-mapping queue served for the mapping matrix generation. To find the optimal mapping, we used information from the adjacent post-mapped subdomain.

Two well center elements in the adjacent subdomains were elected – $\mathbf{X}'_{N_{adj}(B_{i-1,j}),1}$ and $\mathbf{X}'_{N_{adj}(B_{i-1,j}),2}$ in the figure. $\mathbf{V}'$ is a positional variation indicated as (p,q) $where\ p = 0, \pm 1, \dots, \pm p_{max}$ and $q = 0, \pm 1, \dots, \pm q_{max}$ with p_{max} and q_{max} as arbitrary, but empirically reasonable values. Optimal mapping, which gives the maximum number of $\mathbf{X}' \in \mathbf{B}_{\mathbf{i-1,j}}$ to $\mathbf{X}$ matches is found among matrices, which transform $\mathbf{X}'_{Loop,k} \in \mathbf{X}'_{N_{adj}(B_{i-1,j};k)} + \mathbf{V}'$ to $\mathbf{M}\,\mathbf{X}'_{\mathrm{N_{adj}(B_{i-1,j;k})}}$, where k=1, 2 and $\mathbf{M}$ is the mapping of a subdomain where $\mathbf{X}'_{\mathrm{N_{adj}(B_{i-1,j;k})}}$ is included. Here, the term '$\mathbf{X}' \in \mathbf{B}_{\mathbf{i-1,j}}$ to $\mathbf{X}$ matches means that the distance between $\mathbf{M}\,\mathbf{X}'$ and $\mathbf{X}$ is smaller than the empirical threshold value. The iteration is finished when there is no subdomain to be mapped in the pre-mapping queue. After the iteration process, linkage between CCD pixels and well centers is created based on the local mapping matrices through the whole subdomains. For each subdomain, $\mathbf{X} \in \mathbf{A}_{\mathbf{i,j}}$ is transformed by $\mathbf{M}_{i,j}^{-1}$, the nearest $\mathbf{X}' \in \mathbf{B}$ is found and we can define $\mathbf{X}$ is mapped to $\mathbf{X}'$.

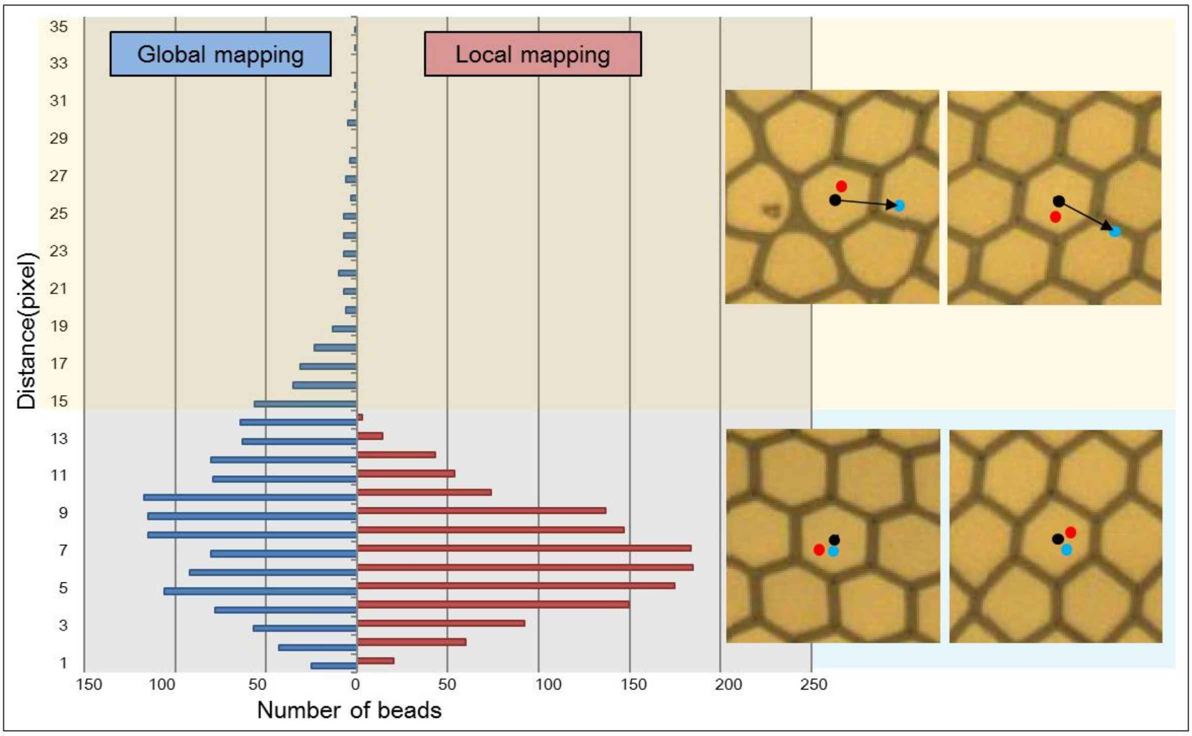

Supplementary Figure 22. Distribution plot of distance between well center and mapped optical signal location. While our local mapping algorithm locates most of optical signals to inside the corresponding well, conventional two-dimensional transformation mislocates 17.5% of the total population. The mislocated populations are not simply indicating loss of available beads. They were mapped into adjacent incorrect wells, reducing the reliability of the entire mapping dataset.

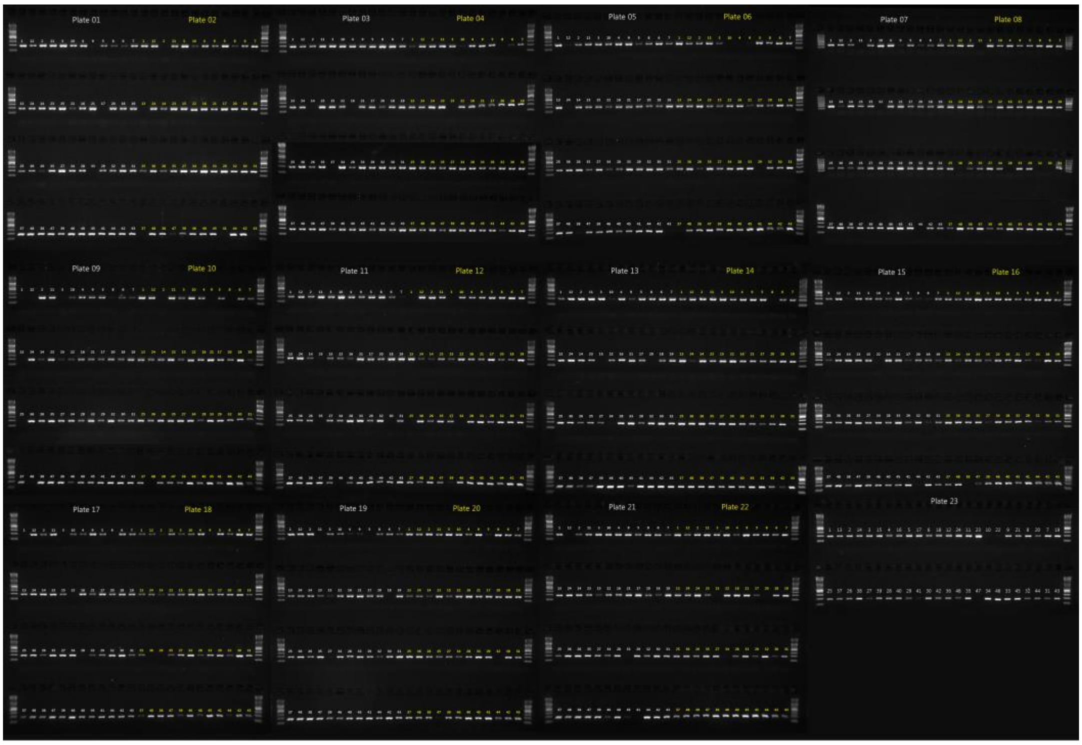
Plate 01
Plate 02
Plate 03
Plate 04
Plate 05
Plate 06
Plate 07
Plate 08
Plate 09
Plate 10
Plate 11
Plate 12
Plate 13
Plate 14
Plate 15
Plate 16
Plate 17
Plate 18
Plate 19
Plate 20
Plate 21
Plate 22
Plate 23

(A)

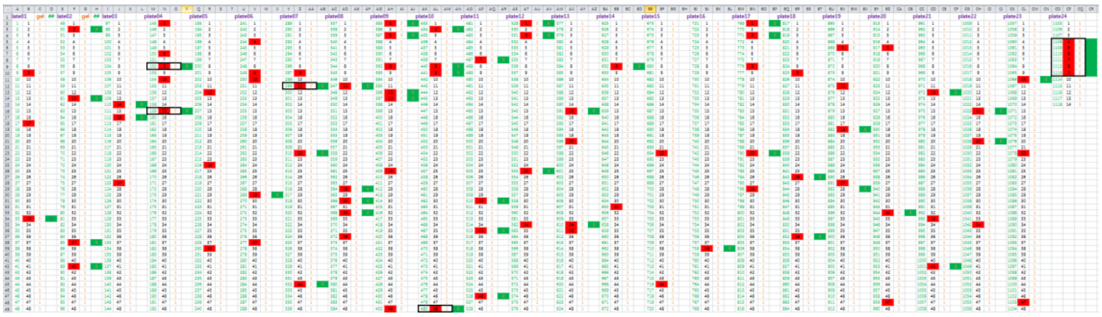

(B)

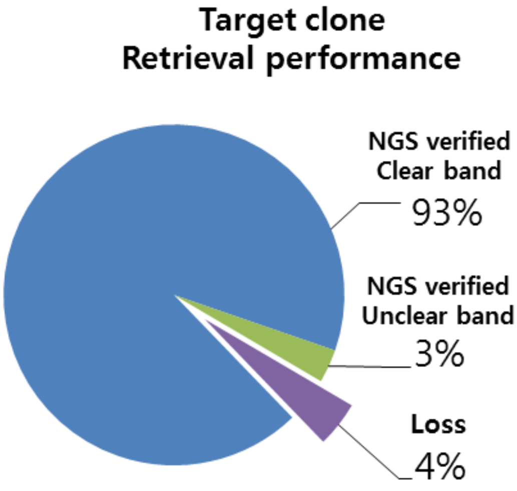
Target clone
Retrieval performance
NGS verified
Clear band
93%
NGS verified
Unclear band
3%
Loss
4%

(C)

Supplementary Figure 23. Target clone retrieval performance of the pulse laser system. (A) Gel image of the PCR product of 1,118 retrieved beads. (B),(C) Target clone retrieval performance of the pulse laser system. The diffusion-like local mapping algorithm provides real-world locations of the 1,108 target clones on the sequencing substrate. The beads were separated and amplified in the individual PCR tube. Each bead retrieval process was confirmed by microscopy. In total, 96% of the contents were NGS-verified, with the 4% loss coming from PCR products with unclear bands

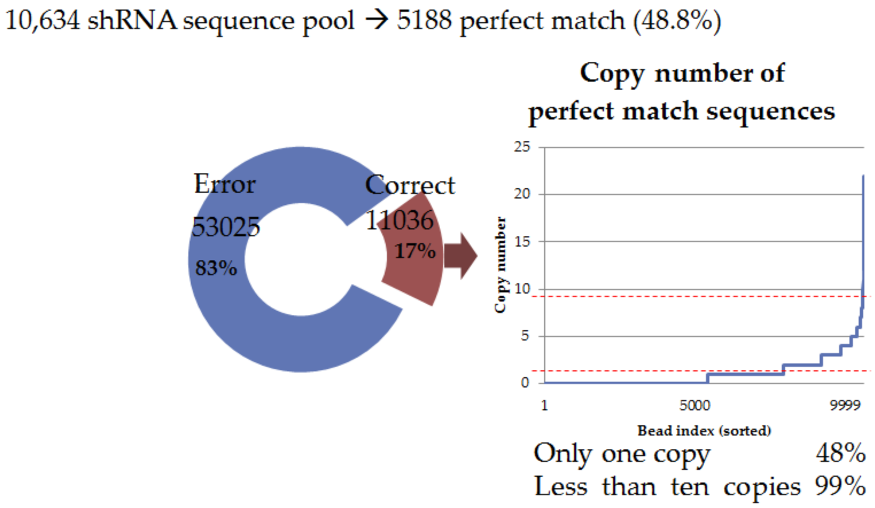

Supplementary Figure 24. 454 NGS result of 10,634 pool sequence. Among 64,061 number of library reads, perfect parts occupy only 17 % (11,036 reads, 5,188 sequences) of total read number.

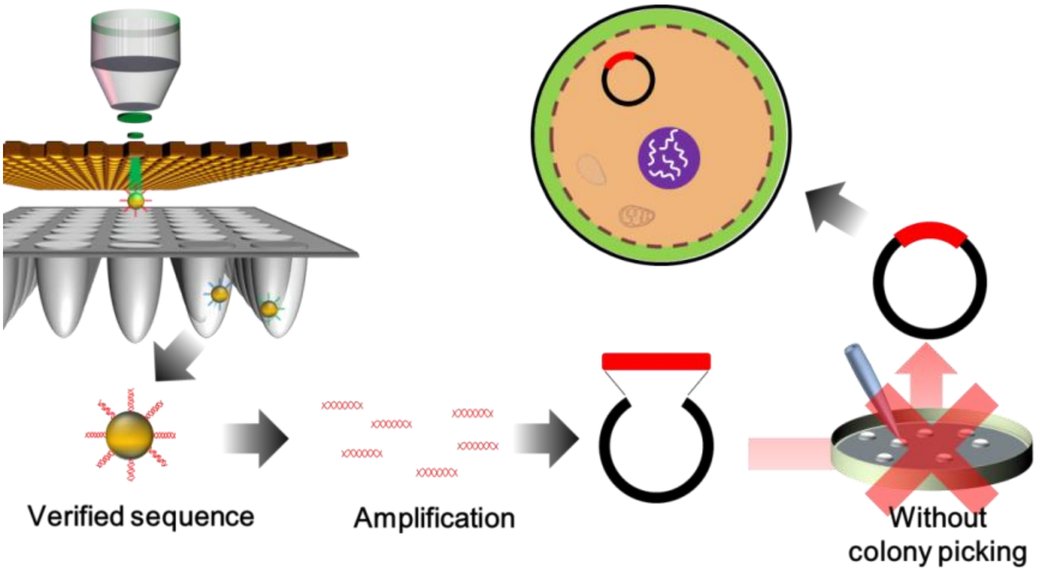

Supplementary Figure 25. Conceptual diagram of direct clone-and-use.

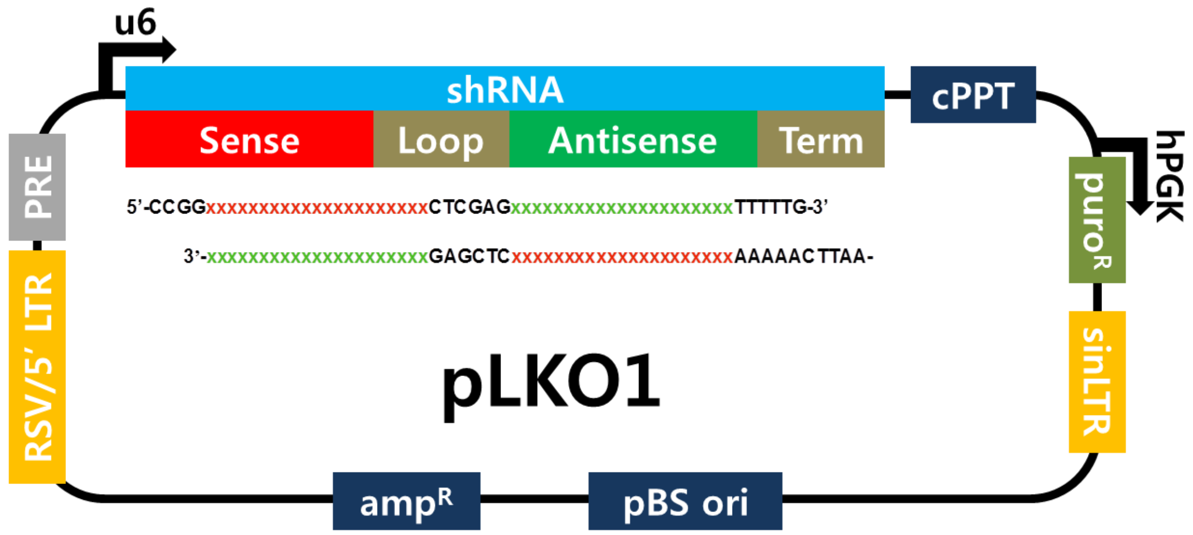

Supplementary Figure 26. Structure of pLKO1 expression vector [2, 3, 4]

TGCAAACTTAGTGCTACATCCTCGAGGATGTAGCACTAAGTTTGCAATTTTT	reference
TGCAAACTTAGTGCTACATCCTCTAGGATGTAGCACTAAGTTTGCAATTTTT	#19
TGCAAACTTAGTGCTACATCCTCGAGGACGTAGCACTAAGTTTGCAATTTTT	#20
TGCAAACTTAGTGCTACATCCTCTAGGATGTAGCACTAAGTTTGCAATTTTT	#35

Supplementary Figure 27. Single base mismatch of Sanger sequencing results of cloning

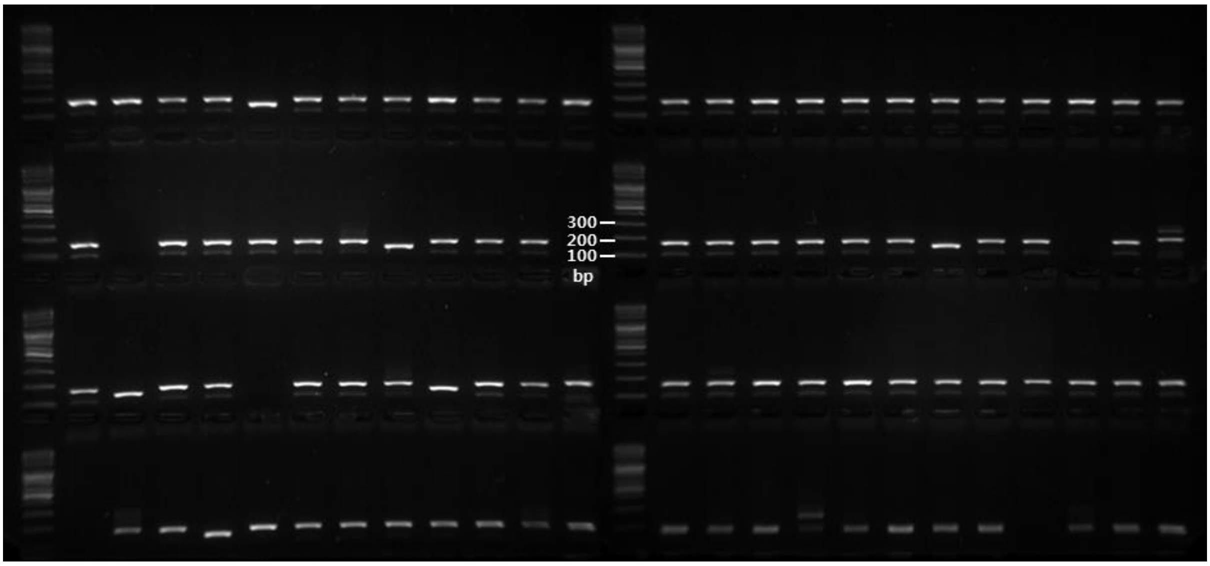

Supplementary Figure 28. Gel image of colony PCR.

Supplementary Notes

Supplementary Note 1 - Cost comparison references

<u>Cost references:</u>

Glass column support (200bp, $0.4/bp from IDT, ultramer synthesis)

DNA microarray (200bp, $2500, custom array)

Commercially available cassettes (gBlock, 500bp, over $100 each)

Sanger sequencing preparation ($3 for TOPO cloning, incubation, seeding or PCR)

Sanger Sequencing ($3 each)

PCR ($0.5/rea)

454 GS FLX+ ($4,000)

Primer pair synthesis ($3 for 15~20mer × 2)

<u>Synthesis cost</u>

Column : ultramer $80, 10,000 ea.	=$800,000
gblock : $100, 4,000ea.	=$400,000
Microarray	=$2,500
Dial-out PCR	=$2,500
Sniper	=$2,500

<u>Error reduction cost</u>

Assume that average perfect part ratio of column and microarray synthesis is 50% without population bias. The necessary number of colony picking and accordant Sanger sequencing should be 2× for column and 200,000× (according to Supplementary Note 2) of their original species number.

Column : Sanger sequencing+preparation $6, 2 times each, 10,000ea.	= $120,000
gblock : Sequence verified commercial product	= $0
Microarray : Sanger sequencing+preparation $6, 200,000 times	= $360,0000
Dial-out PCR: primer $3, 10,000ea, PCR $0.5, 10,000ea.	= $35,000
Sniper: 454 GS FLX+ $4,000, PCR $0.5/rea, 10000ea.	= $9,000

Supplementary Note 2 - Throughput comparison

Theoretical minimum number of clones

Conventional colony picking separation and identification process totally relies on probability. The probability (P) that a given unique DNA sequence is present in a collection of N transformant colonies is given by the following expression [5]

$$P = 1 - (1 - f)^N, \quad (1)$$

where 'f' is the fraction of total genome.

According to this probability function, we conducted a simple calculation to find the minimum amount of colony-picking, taking into account the arbitrary amplification bias and synthesis error.

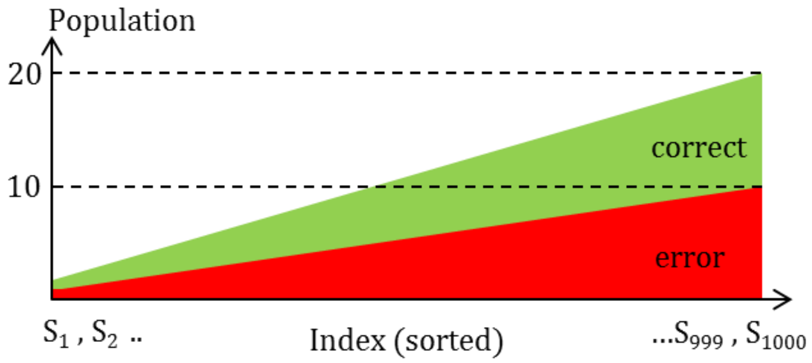

Plot 1. Virtual complex pool constitution including 10x amplification bias and 50% synthesis error.

Assume a hypothetical DNA library containing 1000 individual sequences with a 50% synthesis error rate and linear amplification bias (10×) as shown in the plot 1. After N times of colony picking, the selection probability of each sequence can be described as

$$\begin{aligned} P_{s1} &= 1 - (1 - f_1)^N \\ P_{s2} &= 1 - (1 - f_2)^N \\ &\vdots \\ P_{s1000} &= 1 - (1 - f_{1000})^N, \end{aligned} \quad (2)$$

where $f_n = \dfrac{2 + \frac{18}{999} \times (n-1)}{10000}$ is the relative population of each content.

Thus, the total probability of getting all 1000 contents with N trials is $\prod_{n=1}^{1000} P_{sn}$.

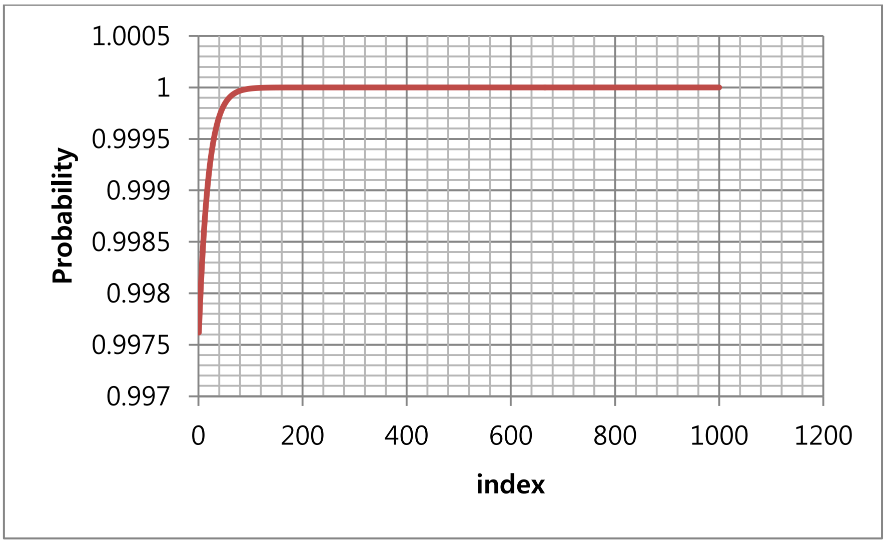

Plot 2. Selection probability plot of 1000 individual sequences (N = 30,197).

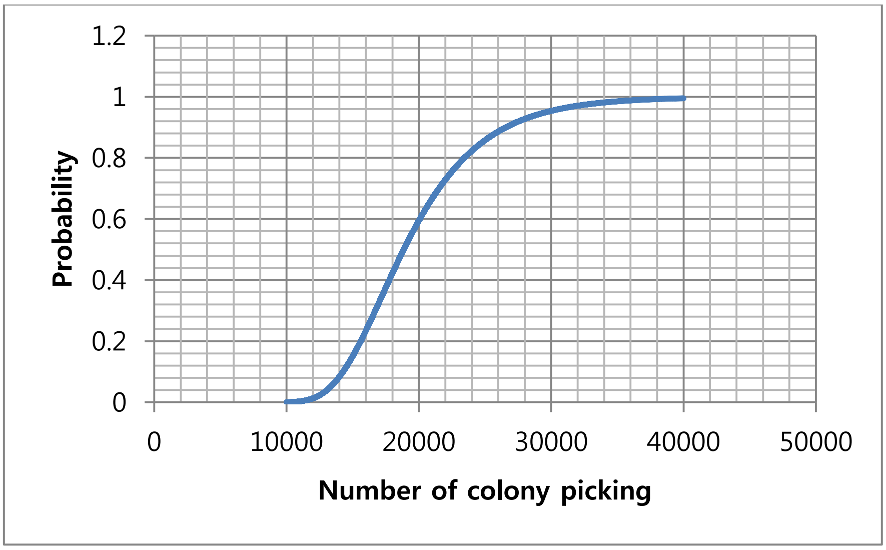

Plot 3. Total probability of getting all 1000 contents versus amount of colony picking.

As shown in the plot 3, 95.6% of 1000 sequence can be recovered when N reaches 30,197, which is almost 30 times more than the number of the original library content. When we consider the 50% error rate, N doubles. To evaluate the effect of amplification bias and synthesis error, we directly compared the selection probability with or without bias and error factors.

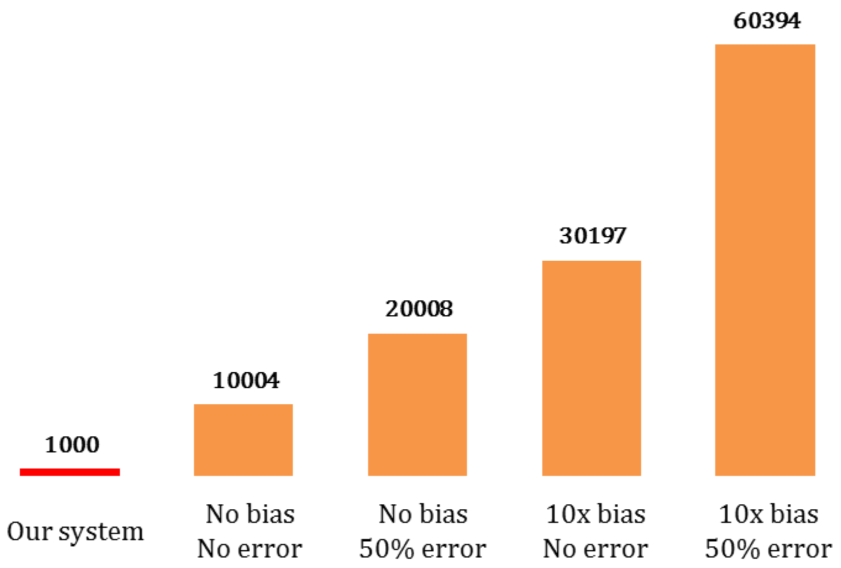

Plot 4. Minimum number of colony picking and sequencing with or without bias and error.

Iterative microarray synthesis and clonal separation do not significantly decrease the necessary amount of colony picking and sequencing. As depicted in Plot 3, 23,438 times of colony picking recovers 80% of the pool contents. Overall, 96% recovery is achievable with additional microarray DNA synthesis, which are responsible for the remaining 20% followed by 3,443 times (totally 26,881) of colony separation and capillary sequencing.

Supplementary Note 3 - Technical comparison ('Shot-gun synthesis' *vs.* 'Sniper Cloning')

In this note, we would like to discuss comparative advantages of 'Sniper Cloning' based on the empirical shortcomings of 'Shot-gun DNA synthesis' approach.

Recently our group conducted selective amplification experiment using 1000 by 1000 (total of 10^6) barcode primer and accordant microarray derived DNA mixture pool. In this result, we observed considerable portion of mixtures in selective amplification. Some of them even had no amplified products (unpublished results). We think that those unwanted results come from the complexity of mixed pool of DNA and incomplete orthogonality between primer pairs or primers and pool sequence. This is expected from the examples of target capture probes, not successfully amplifying specific regions in complex mixture. Therefore, extra processes should be carried out before NGS followed by dial-out PCR to reduce the complexity of mixed pool into appropriate level. Meanwhile, we found that there were more barriers in the use and preparation of primer pairs. The preparation of primer pairs maintaining orthogonality between primers or between primers and pool contents required very picky design rules, long time and great expense. In spite of the efforts, we only obtained non-uniformly amplified products with some missing parts due to the difference of primer sequence specific amplification efficiency, forming hairpins, or forming dimers. In some cases, when we tried to separate more than 1,000 species from mixture, we ran out of mixture pool (1 μl/reaction), leaving no choice but to amplify mixed pool with universal primer bearing the risk of PCR bias.

'Sniper Cloning' technology physically separates and utilizes NGS verified megaclones from the sequencing substrate using focused pulse laser and custom made, open source mapping algorithm suggests this approach's several advantages over previous 'Shot-gun synthesis' (or Dial-out PCR).

1) **Universal primer for amplification:** Physical separation of metaclones allows us to use optimum universal primer for amplification. This eliminates huge side-effects caused by primer pair usage in 'Shot-gun synthesis'

2) **Stability:** Regardless of the contents of microarray derived mixed pool, 'Sniper Cloning' separates and retrieves every oligonucleotide species that are on NGS sequencing substrate. The term 'Sniper' implies target identification and retrieval,

consecutively. Thus, when the target species has low copy number in NGS process due to any reason, 'Sniper Cloning' can accurately retrieve target sequence in a stable and high-throughput manner without any additional process. It seems difficult to distinguish sequencing error and synthesis error in present state. However, we expect that the sequencing accuracy of NGS technology would be augmented. It is obvious that the consensus sequencing has more advantages over single-pass methods. However, since the previous arts and our 'Sniper Cloning' occupy in an identical position in the process of mass production as an error reduction method, consensus sequencing can be applied to both previous arts and our method. For example, barcode sequences generated in synthesis process would enable us to decouple sequencing error by observing same barcode sequence in the sequencing data for the retrieval of target sequence. This approach combines the accuracy of barcode tagging approach with the greater retrieval stability of 'Sniper Cloning', improving on the best aspects of each approach.

Meanwhile, according to the report from previous research by Matzas et al, the average error rate of post-amplification mainly induced by polymerase incorporation is much higher than that of sequencing or synthesis. Thus, we believe that it is a matter of little importance.

3) **Long term cost reduction:** 'Sniper cloning' needs initial instrument cost to build the system including microscope, pulse laser and motorized stage. But the optical system of 'Sniper Cloning' is very simple and most of bio-labs have commercial microscope. Since we're going to make our custom made mapping algorithm open to the public, it only requires tens of thousand dollars to build and use their own system semi-permanently. There are no huge costs for primer synthesis and robotics or for their combination. When we think about sky rocketing capacity of NGS, it is definitely going to benefit users in the long run in terms of expense.

Scalability and technical potential: Most of current NGS platforms can be divided into two primary categories: involvement of microstructure (microbead) (ex. 454 or Iontorrent); and direct attachment of DNA clusters on the surface of sequencing substrate (ex. Illumina). Our 'Sniper Cloning' system is designed to apply both NGS platforms. Main manuscript describes the first version of 'Sniper Cloning' with the help of the 454 NGS platform. DNA carrying microbead in the 454 sequencing plate can be separated by small force. We deliver such force with radiation pressure of focused pulse laser. Since this non-contact phenomenon occurs

from UV to NIR spectral region, one can choose specific wavelength to avoid DNA damage or to increase substrate transmission. For example, 454 sequencing substrate is transparent for both 532nm (green) and 1064nm (NIR), while silicon wafer based Iontorrent chip has high transmittance on 1064nm laser. The second version of 'Sniper Cloning' is for platforms without microstructure such as those of Illumina. There are no changes in hardware between version 1 and 2 except the output power of pulse laser (double the power level, same instrument). The difference is just for the users' convenience. To retrieve sequence verified DNA from the substrate of Illumina sequencer, we use plasma development pressure caused by pulse laser ablation of substrate. As shown in Supplementary Fig. 2 and Fig. 6, we intentionally focused laser pulse slightly above (inside) the surface of substrate. High energy of focused laser pulse turns small volume of substrate into plasma status. The force of those expansion pushes target substrate region together with target DNA sequence to PCR tube.

Previous literature already showed the possibility of mapping Illumina clusters and utilizing them in a small region of the flow cell[2]. We expect that our mapping algorithm can be generalized to this approach by eliminating non-linear imaging distortion induced by tapered fiber or line scanning imaging methods, which would enable whole flow cell utilization. Also, it is well known that the size of ablation spot can be reached to sub-micrometer level when it comes to shorter pulse laser such as picosecond or femtosecond pulse laser instead of the nanosecond pulse laser. Therefore, we believe that the issue about ablation spot size seems solvable with proper hardware upgrade.

\

Supplementary Note 4 - Radiation pressure

To avoid both system damage and retrieval failure, the appropriate radiation force should be exerted on the backside of the target bead-containing well. In our system, approximately 50μJ/pulse energy is sufficient for bead retrieval without damaging the optics of the microscope, CCD camera, sequencing substrate and DNA sequence attached on the surface of the bead. The amount of impulse can be calculated as follows [6]:

$$\text{Radiation pressure}: \frac{S(t)}{c} \tag{3}$$

where, $S = \frac{Energy_{total}}{Area \cdot Time}$ and velocity of light c.

$$Energy_{total}: 50\mu\text{J/pulse} \tag{4}$$

$$\text{Area}: \pi(15\mu m)^2 \tag{5}$$

$$\text{Time: 7ns} \tag{6}$$

Hence, within 7ns, the target bead will get a radiation force of 0.25μN which totals a 1.67fNs impulse that is transferred to the bead from a focused single pulse laser shot.

Supplementary Note 5 - Quality improvement

The quality of initial pool of DNA is very hard to measure, unless someone has ideal single oligonucleotide sequencer and counter. Hence, despite of the amplification bias and sequencing error, previous researches commonly use NGS analysis to indicate the quality (error rate) of initial DNA pool. For the error rate calculation of final pool, as one can see in previous literature, it is reasonable to eliminate reads that contain deletions because major deletion reads come from the sequencing error. However, when the same calculation method is applied to initial pool, it induces critical over-estimation. The major portion of deletion reads comes from the synthesis error which is much larger than the sequencing error. Thus, equivalent error rate estimation can be drawn by multiplying deletion sequencing error effect to perfect match population. From the Fig. 4 in the main manuscript, the empirical value of deletion error effect is 95.4/85.1=1.12

then the effective perfect read increases to 12781×1.12 = 14314

and accuracy increases from 19.95% to 22.3%

as a result, estimated error rate becomes 1 in 69.9bp

[Filtered pool]

Accuracy: 95.7%

Estimated error rate: 1 in 2,367bp

Supplementary Methods

Pulse laser bead retrieval system

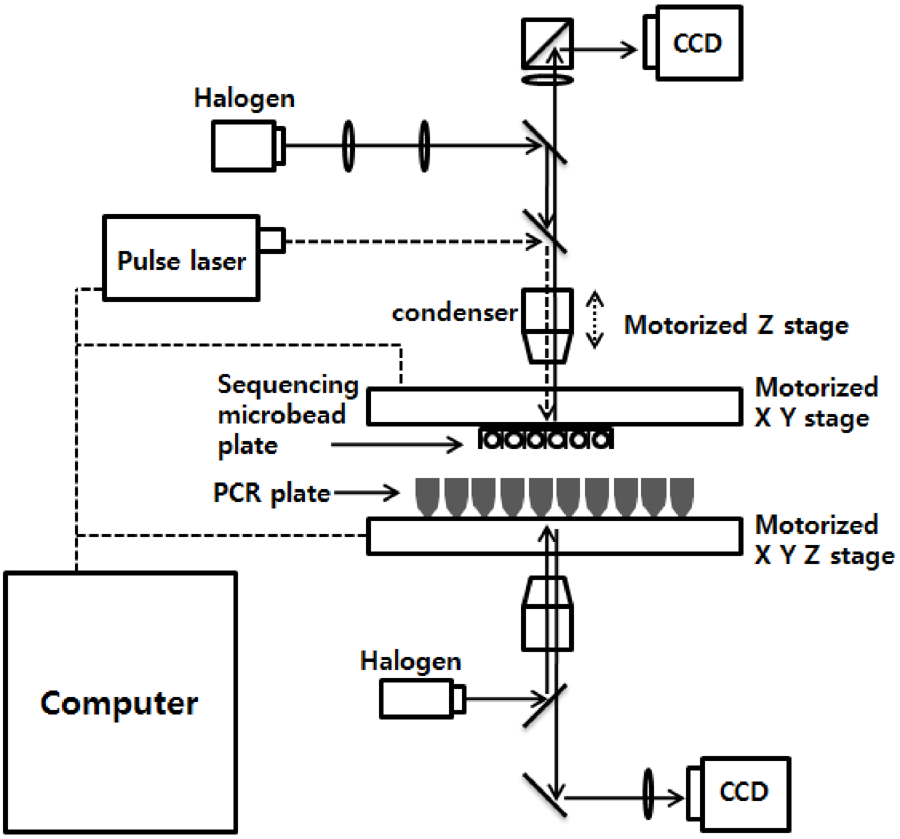

Schematic diagram of the pulse laser bead retrieval system. Pulse laser, CCD camera and two motorized stages are controlled by a personal computer with self-made Labview software. The upper part of the system, a commercial inverted microscope and a motorized stage, are hung upside down where the direction of the radiation force is identical to that of gravity. We constructed the whole system, aside from the personal computer and pulse laser power supply, on an anti-vibrational optical table.

The system was composed of the following:

1. Two CCD cameras (Guppy PRO F-146C, ALLIED) for scanning and imaging the 454 sequencing plates and retrieval bead observation.
2. Two motorized stages, top one (SCAN IM120X100, MärzhäuserWetzlar) for the sequencing plate and bottom one (SCAN 100X100 ,MärzhäuserWetzlar) for the PCR plate.
3. One inverted microscope (IX71, Olympus) with a 10X objective lens.
4. One Compact Q-Switched Nd:Yag laser system (Minilite, Continuum)
   (28mJ at 1064nm, 12mJ at 532nm, 4mJ at 355nm, 2mJ at 266nm, Repetition rate: 1-15Hz)
5. One personal computer for controlling the pulse laser and motorized stages with Labview.

pLKO1 **vector cloning protocol**

- Vector: TRC2_pLKO-puro (50ng/µl)
- Restriction enzyme digestion (vector : insert = 1:10)

Reaction conditions for pLKO1 vector cloning

d.w	NEBuffer4	10X BSA	AgeI & EcoRI	Insert (184bp)	Vector (7484bp) : TRC2_pLKO-puro (50ng/µl)
11.92	2 µl	2 µl	1µl each	1.08 µl of 130520_p8_7_gp	1 µl
11.19	2 µl	2 µl	1µl each	1.81 µl of 130520_p8_10_gp	1 µl
11.83	2 µl	2 µl	1µl each	1.17 µl of 130520_p8_18_gp	1 µl
11.81	2 µl	2 µl	1µl each	1.19 µl of 130520_p8_31_gp	1 µl

➔ Incubation at 37℃ for 2 hours.

- PCR purification

➔ Elution with 20 µl of distilled water

- T4 DNA ligation

➔ Added 2 μl of T4 DNA ligation buffer and 1 μl of T4 DNA ligase into each tube.

➔ Incubation at 16℃ overnight

- Used 10 μl of ligated DNA for transformation.

Supplementary References

1. Faruqi AR, Henderson R, Subramaniam S. Cooled CCD detector with tapered fibre optics for recording electron diffraction patterns. *Ultramicroscopy* **75**, 235-250 (1999).

2. Moffat J, *et al.* A lentiviral RNAi library for human and mouse genes applied to an arrayed viral high-content screen. *Cell* **124**, 1283-1298 (2006).

3. Paddison PJ, *et al.* A resource for large-scale RNA-interference-based screens in mammals. *Nature* **428**, 427-431 (2004).

4. Root DE, Hacohen N, Hahn WC, Lander ES, Sabatini DM. Genome-scale loss-of-function screening with a lentiviral RNAi library. *Nat Methods* **3**, 715-719 (2006).

5. Clarke L, Carbon J. A colony bank containing synthetic Col El hybrid plasmids representative of the entire E. coli genome. *Cell* **9**, 91-99 (1976).

6. Kleiman ZY, Golovizn.Gi. Measurements of Light Pressure and Pulse Energy of Laser Radiation. *Izv Vuz Fiz+*, 48-& (1969).
